# HIV capsid inhibitors: Mechanisms, resistance, and therapeutic advances

**DOI:** 10.1371/journal.ppat.1014361

**Published:** 2026-07-10

**Authors:** Mohamed Mahdi, Botond Lakatos, János András Mótyán, Gyula Hoffka, József Tőzsér

**Affiliations:** 1 Department of Biochemistry and Molecular Biology, Faculty of Medicine, University of Debrecen, Debrecen, Hungary; 2 Department of Infectology, Faculty of Medicine, University of Debrecen, Debrecen, Hungary; 3 Department of Hematology and Infectious Diseases, Departmental Group of Infectious Diseases, Semmelweis University, Budapest, Hungary; 4 Department of Chemistry, Lund University, Lund, Sweden; Fred Hutchinson Cancer Center, UNITED STATES OF AMERICA

## Abstract

The capsid (CA) of the human immunodeficiency virus (HIV) has emerged as a critical therapeutic target owing to its essential roles in viral replication, nuclear import, and integration. Capsid inhibitors (CIs) disrupt these processes by binding to highly conserved structural interfaces, offering a novel mechanism distinct from enzyme-targeting antiretrovirals. In this review, we summarize the molecular architecture of the HIV-1 CA and its interactions with host factors, and we compare key structural and functional differences between HIV-1 and HIV-2. We provide an overview of established and investigational CIs, explore the resistance-associated mutations, their structural basis, and their impact on inhibitor potency, alongside insights into cross-resistance patterns. Special emphasis is placed on lenacapavir, exploring data from pivotal trials and emerging applications in both treatment and prevention, including long-acting pre-exposure prophylaxis (PrEP). Moreover, we highlight future perspectives, including the need for global surveillance of CA polymorphisms, strategies to overcome resistance, and challenges in accessibility and cost-effectiveness in resource-limited settings. Collectively, CIs represent a transformative addition to the HIV therapeutic arsenal, though their optimal deployment requires careful consideration of efficacy, resistance, and implementation barriers.

## 1. Introduction

At the time of writing this review, more than 40 million people (37–45.6 million) are living with HIV, with 1.3 million (1.0 –1.7) new infections occurring in 2024 alone, according to the World Health Organization (WHO) [1]. The ongoing global epidemic of HIV continues to pose a significant threat to public health worldwide, necessitating sustained efforts in the development of effective treatment strategies [2].

There are two main types of HIV: HIV-1 and HIV-2, both of which share the same basic genetic organization and replication strategy; however, they differ in transmissibility, geographic distribution, and clinical course [3,4]. HIV-1 drives the global HIV/ acquired immunodeficiency syndrome (AIDS) pandemic, infecting tens of millions worldwide and accounting for the vast majority of cases due to its high transmissibility and rapid disease progression [5,6]. In contrast, HIV-2 remains largely localized to West Africa, although there is evidence of gradual spread beyond its traditional geographic boundaries [7,8].

Both HIV‑1 and HIV‑2 are classified within the subfamily *Orthoretrovirinae* of the family *Retroviridae* [3]. Comparative phylogenetic studies indicate that these viruses arose from cross‑species transmissions of simian immunodeficiency viruses (SIVs), with HIV‑1 originating from SIVcpz in chimpanzees and HIV‑2 from SIVsmm in sooty mangabeys, likely through several independent zoonotic events [9,10]. Molecular clock analyses suggest that these spillover events may have taken place several centuries in the past. HIV‑1 was first isolated in 1983 from a patient showing early signs of AIDS, while HIV‑2 was first identified in 1986 in West African individual presenting with AIDS‑related symptoms, establishing it as a distinct but evolutionarily related lineage [11,12].

HIV-2 exhibits strong genomic similarity to HIV-1. Aside from differences in nucleotide sequence and the presence of distinct accessory genes; such as viral protein X (Vpx) in HIV-2 and viral protein U (Vpu) in HIV-1, along with variations in the envelope proteins and their functional properties, both viruses display a comparable overall genomic architecture [3]. From a clinical perspective, while infection with either virus can eventually lead to AIDS, HIV-2 is characterized by lower infectivity and reduced pathogenicity. Studies indicate that individuals infected with HIV-2 are more likely to remain long-term nonprogressors, as progression from the initial infection to AIDS is typically much slower and more protracted [13,14].

Building on these similarities, important distinctions in HIV-2 biology merit attention, particularly in viral entry, replication, and latency regulation. HIV-2 engages CD4 and co-receptors CCR5/CXCR4 alongside a broader array (such as CCR1, CCR2b, CCR3, CCR8, CXCR1, GPR1, apj, and us28), enabling CD4-independent infection *in vitro*, although this was not found to enhance pathogenicity [15–17]. Replication kinetics differ markedly, with HIV-2 exhibiting lower plasma/DNA loads, an acute post-infection surge, and extended latency versus HIV-1’s steadier output [15], potentially tied to larger, structurally complex long-terminal repeats (LTRs) that feature duplicated TAR elements and distinct promoters [18]. These characteristics underpin HIV-2’s slower progression to AIDS, and in dual infected individuals, suppression of HIV-1 via transcriptional interference [19,20]. Moreover, HIV-2 elicits more polyfunctional CD4+ and CD8+ T-cell responses, producing multiple cytokines along with broader neutralizing antibody response directed against primary isolates, compared to HIV-1. These robust immune features of the infection likely also contribute to its attenuated disease course [4]. Overall, despite the key differences between the two viruses, research on HIV-2 remains limited, perhaps the scarcity of epidemiological statistics and prevalence data may partly explain why it has received far less scientific attention than HIV-1.

AIDS is the late, symptomatic stage of untreated HIV infection, defined by severe depletion of CD4+ T cells and the subsequent collapse of adaptive immune function [21]. As viremia persists in the absence of effective antiretroviral therapy (ART), progressive T-cell loss impairs cellular and humoral immunity, rendering individuals susceptible to opportunistic infections; such as *Pneumocystis jirovecii* pneumonia, disseminated mycobacterial disease, and invasive fungal infections, as well as certain malignancies such as Kaposi’s sarcoma and B-cell lymphomas [22]. Clinically, AIDS is diagnosed either when CD4+ T-cell count falls below the 200 cells/μl threshold, or when an infected individual develops one or more AIDS-defining illness; however, combination antiretroviral therapy (cART) regimens can suppress viral replication, preserve immune function, and prevent progression to AIDS in most treated patients [23].

Besides monoinfections, dual HIV‑1 and HIV‑2 infection (HIV‑D) has long been described, but its impact on clinical prognosis remains incompletely defined, supported by limited cohorts [19,24,25]. While recent population level estimates remain limited, HIV‑D is most prevalent in West Africa, where both viruses are endemic. Current evidence indicates that dually infected individuals tend to have lower HIV‑1 viral loads and a slower rate of progression to AIDS compared with those monoinfected with HIV‑1. Proposed mechanisms for this relative attenuation include altered receptor usage, suppression of T-cell activation, overexpression of beta‑chemokines, and the development of cross‑reactive humoral and polyfunctional T helper and cytotoxic T‑cell responses directed against both viruses [20,24,26,27].

Over the past four decades, substantial progress has been achieved in the management of HIV infection, transforming what was once a rapidly fatal illness into a chronic condition suitable for long-term management [28]. This remarkable shift is largely attributed to the development and implementation of highly active antiretroviral therapy (HAART), which has evolved from the era of monotherapy with NRTIs to the current standard of antiretroviral therapy also known as cART [29]. HAART, introduced in the mid-1990’s, relied on combining three or more antivirals from at least two different classes to achieve durable viral suppression, while modern cART builds on this principle but is now characterized by integrase strand transfer inhibitors (INSTIs); such as dolutegravir (DTG) and bictegravir (BTG) as preferred anchor agents, owing to their rapid virologic suppression, high genetic barrier to resistance, and generally favorable toxicity profile. These regimens have substantially reduced the incidence of treatment-related adverse events and improved adherence compared to older HAART protocols, that relied heavily on PIs and more toxic NRTIs [30,31].

Nonetheless, challenges related to drug toxicity and resistance persist in the current cART era as well. Long-term treatment can still lead to renal impairment, bone mineral loss, dyslipidemia, neuropsychiatric side effects among other, necessitating careful regiment selection and monitoring [31]. Moreover, the emergence of DTG-associated mutations in individuals with prior treatment experience or suboptimal adherence remains a concern [32].

In parallel, the development of pre-exposure prophylaxis (PrEP), mostly based on tenofovir-based regimens, has shifted the paradigm from treatment-only to prevention-integrated strategies, reducing new infections in high-risk populations given that adherence is maintained [33]. Taken all together, these advances underscore that while cART has markedly improved safety and efficacy over earlier HAART, optimal management still requires ongoing monitoring of toxicity, resistance, and long-term adherence.

The introduction and advancement of ART have dramatically improved the prognosis for individuals living with HIV in the past 30 years [34], leading to significant increases in life expectancy and enhanced quality of life; however, CD4+ cell count at the initiation of ART remains a critical determinant of life expectancy [35]. This evolution underscores the critical role of pharmacological interventions in combating this persistent viral threat. The continuous pursuit of optimized therapies, including CIs, remains paramount in the face of the virus’s adaptability and the aspiration for improved patient outcomes.

The therapeutic landscape for HIV encompasses seven classes of antiretroviral drugs, each targeting a distinct stage of the viral life-cycle. These include i) entry inhibitors; including fusion inhibitors and CCR5 antagonists, which prevent the virus from entering host cells; ii) post-attachment inhibitors, which block post-binding steps of viral entry, iii) capsid inhibitors (CIs), which disrupt the formation or disassembly of the viral capsid; iv) nucleoside/nucleotide reverse transcriptase inhibitors (NRTIs), and v) non-NRTIs (NNRTIs), which interfere with and inhibit reverse transcriptase; vi) integrase strand transfer inhibitors (INSTIs), which prevent viral genomic integration; and vii) protease inhibitors (PIs), which block the viral protease enzyme needed for the formation and maturation of new viral particles [36].

While each of these drug classes has contributed significantly to the management of HIV infection, they are also associated with limitations; such as potential toxicities, the development of drug resistance, and in some cases, complex dosing regimens [37], underscoring the urgent need for innovative therapeutic strategies, and the development of agents with improved efficacy, safety profile, tolerability, and convenience, in order to improve adherence.

Once HIV diagnosis is confirmed, current guidelines recommend that antiretroviral therapy be started immediately for all people living with HIV, irrespective of their WHO clinical stage or CD4 cell count, with the exception of certain conditions such as cryptococcal meningitis, in which case treatment should be deferred to avoid the development of immune reconstitution inflammatory syndrome (IRIS) [38]. The preferred initial antiretroviral therapy (ART) regimen in adults consists of a nucleoside reverse transcriptase inhibitor (NRTI) backbone of tenofovir disoproxil fumarate (TDF) or tenofovir alafenamide (TAF) plus lamivudine (3TC) or emtricitabine (FTC), combined with dolutegravir (DTG), absent contraindications [38]. Current guidelines generally apply the same ART approach to HIV‑2 and HIV-D, adjusted according to local drug‑availability and HIV‑2‑specific considerations [38,39].

The HIV capsid (CA) protein forms the conical core that encloses the viral genome and associated enzymes, playing essential roles throughout the viral life-cycle. CA orchestrates early events such as core stability, reverse transcription, trafficking toward the nucleus, and nuclear import of the pre-integration complex, while also contributing to late-stage processes including virion assembly and maturation [3]. Because CA function is tightly coupled to both viral replication and evasion of host restrictions factors, it represents a structurally and mechanistically compelling target for interference, therefore, targeting the HIV CA is viewed as a central node for next-generation antiviral strategies. In light of this importance, this review is dedicated to a comprehensive examination of capsid biology and inhibition. We summarize the molecular architecture of the HIV CA protein and its dynamic interactions with host cellular factors, while also comparing key structural and functional distinctions between HIV-1 and HIV-2. Furthermore, we provide a detailed discussion of the history and development roadmap of CIs, outlining their advantages and potential limitations, identifying critical CA residues involved in drug interactions, and evaluating the impact of resistance-associated mutations. Moreover, we compare and analyze the potential applicability of these inhibitors for the treatment of HIV-2, a less prevalent but clinically relevant HIV type.

## 2. HIV capsid

The HIV-1 CA core encapsulating the viral RNA and vital enzymes is assembled from CA monomers, and is indispensable for viral replication, facilitating processes such as reverse transcription, nuclear import, and integration of the viral genome into the host cell’s DNA [40–42]. The synthesis of the CA protein involves the translation of the viral *gag* gene to Gag polyprotein, followed by proteolytic cleavage by the HIV protease. This cleavage generates the mature CA protein, which assembles into a conical structure [43].

### 2.1 Function of HIV CA

Beyond its structural role, the HIV CA functions as a multifunctional orchestrator throughout the early stages of viral replication [44]. During reverse transcription, the CA core acts as a protective scaffold, cores that are unstable or excessively rigid were found to compromise viral DNA synthesis, indicating that a finely tuned, dynamic stability is essential for successful reverse transcription [45]. The incorporation of inositol hexakisphosphate (IP6) into the HIV-1 CA core was shown to stabilize the CA while maintaining its semipermeable properties [46], enhancing nucleotide permeability through the hexamer pores, and promoting efficient reverse transcription [47]. CA is also central to cytoplasmic trafficking, recruiting microtubule-associated proteins and motor adaptors that guide the viral core toward the nucleus along the cytoskeleton [48]. At the nuclear pore, CA engages with nucleoproteins such as Nup358 and Nup153, facilitating docking and translocation of the reverse transcription complex into the nucleus [49,50]. Inside the nucleus, the CA influences not only the final uncoating step required for integration, but also where integration occurs, though interaction with the host factor CPSF6, whereby CA directs the viral genome toward gene-dense, transcriptionally active regions of chromatin located near nuclear speckles [51].

### 2.2 Structure of the CA

**Fig 1:**
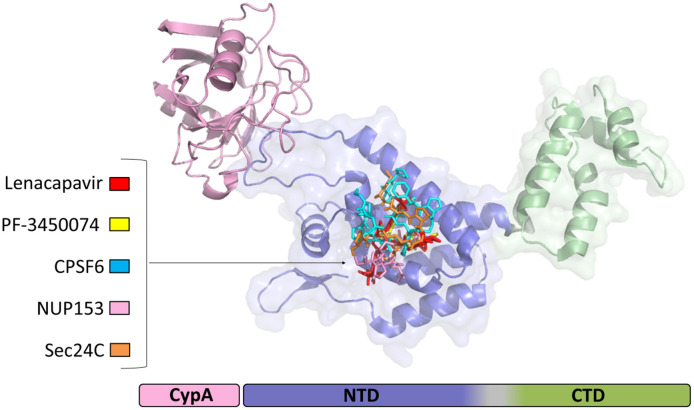
Structure of HIV-1 CA. Structure of full-length HIV‐1 CA complexed with CypA is shown based on crystal structures: (PDB ID: 3NTE) [69] and (PDB ID: 1AK4) [58]. Color code: CypA, purple; NTD, blue; CTD, green. Molecules binding to the hydrophobic pocket are represented based on crystal structures of HIV-1 CA complexed with lenacapavir (PDB ID: 6VKV) [70], PF-3450074 inhibitor (PDB ID: 2XDE) [71], CPSF6 peptide (PDB ID: 4B4N) [72], NUP153 peptide (PDB ID: 4U0C) [73], or Sec24C (PDB ID: 6PU1) [74]. The ligand molecules are shown by stick, and the color code is shown in the figure.

The mature HIV-1 CA consists of 231 amino acid residues. The full-length protein has predominantly α-helical secondary structure, and its tertiary structure consist of two independently folded domains. The N-terminal domain (NTD, 1–145 residues) is considered as core domain, while the C-terminal domain (CTD, 151–231 residues) as dimerization domain, which are connected by a short linker [48,52–55]. The structure of HIV-1 CA is represented in Fig 1. The structurally and functionally important regions of HIV-1 CA are well-characterized, and have already been reviewed previously [40,48,56,57].

One of the functionally important sites is a surface loop of NTD (residues 87–92) [58], which was found to bind the host-derived cofactor peptidyl-prolyl isomerase cyclophilin A (CypA). The incorporation of CypA into HIV-1 particles was found to be advantageous for the formation of infectious virions [59–61]. While it was found to often enhance infectivity, its role is strain-dependent, with some HIV-1 strains showing minimal or no reliance on CypA for infectivity, particularly in cell lines such as HeLa or H9, or with CA mutations such as G89V or A92E [61,62]. It is important to mention that while HIV-2 CA protein does bind CypA, the interaction is weaker and less functionally significant compared to HIV-1 [63,64].

The evolutionarily conserved major homology region (MHR), a 20-residue segment within the CTD spanning residues 153–172, plays a crucial role in maintaining structural integrity and mediating interactions with both viral and host molecules [65]. Both the NTD and CTD contain cleavage sites for HIV-1 protease, and can be proteolytically processed [66], however, the role of the viral protease during the early phase of the viral life-cycle remains controversial [67,68].

Intermolecular interactions between the NTD and CTD of adjacent CA monomers contribute to the structural integrity of both hexameric and pentameric capsomers [75]. This inter-domain interface also forms a conserved hydrophobic pocket that mediates interactions with host cellular cofactors involved in the early stages of infection (Fig 1) [76,77]. Its structural stability and functional versatility, combined with its high sequence conservation across HIV-1 strains, make it an attractive target for antiretroviral drug development [78–80].

### 2.3 Comparison between HIV-1 and HIV-2 CA

**Fig 2:**
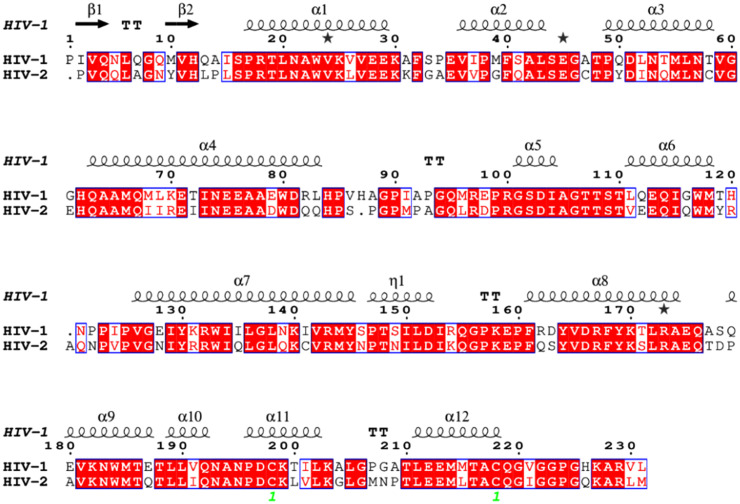
Sequence alignment of HIV-1 and HIV-2 CA. Sequence alignment was performed using ESPript 3.0 online tool [81]. Secondary structural arrangement is indicated based on a crystal structure of full-length HIV-1 CA (PDB ID: 3NTE) [69].

Across HIV-1 groups, amino acid conservation in the HIV CA ranges from 70 to >80%, and while the overall structure of the CA is highly conserved, variability in the secondary structure can be observed between different groups [80]. The full-length CA of HIV-1 and HIV-2 share a 69% overall sequence identity, the sequences of NTD and CTD are 66% and 74% identical, respectively (Fig 2).

**Fig 3:**
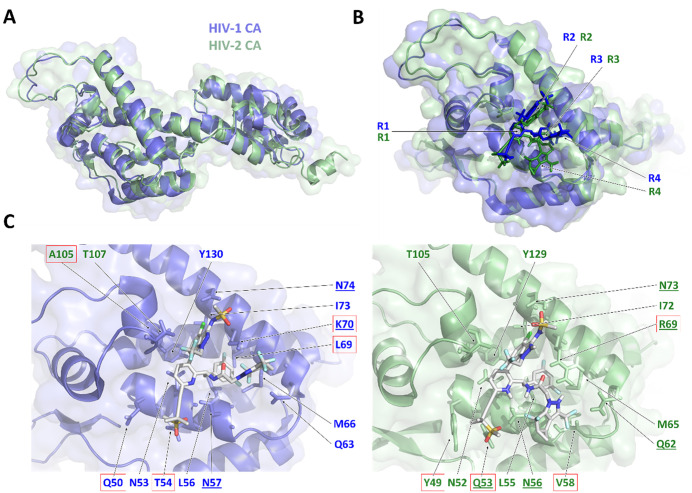
Structure of HIV-1 and HIV-2 CA and binding mode of lenacapavir. **(A)** Structural alignment of HIV-1 and HIV-2 CA. HIV-1 CA is represented based on a crystal structure (PDB ID: 6VKV) [69], while the structure of HIV-2 CA was modeled previously using AlphaFold [82]. **(B)** Comparison of the binding mode of lenacapavir to the NTD of HIV-1 and HIV-2 CA. The enzyme-inhibitor complex is shown based on a crystal structure of HIV-1 (PDB ID: 6VKV) [70], while based on molecular docking for HIV-2 [82]. Lenacapavir is shown by blue and green colors for HIV-1 and HIV-2 CA, respectively. The pyridinium (R1), the indazole (R2), the difluorobenzyl (R3), and cyclopenta-pyrazole (R4) rings of lenacapavir are highlighted in blue and green for HIV-1 and HIV-2, respectively. **(C)** Binding interactions of lenacapavir are shown based on structural analysis of HIV-1 and HIV-2 CA [82]. Interactions are shown only for NTD but not for CTD. Underlined residues contribute to hydrogen-bond formation; otherwise, nonbonded contacts are formed. Residues in red boxes are different or form interactions only in HIV-1 and HIV-2 CA.

Besides sequences, structures of HIV-1 and HIV-2 CA are also highly similar (Fig 3A).

In our previous work, we determined the efficacy of lenacapavir (LEN) against HIV-1 and HIV-2 in a cell culture-based assay [82]. Comparison of the inhibitor-bound complexes revealed that HIV-2 CA also contains a surface pocket that can bind LEN. Docking of LEN to this pocket of HIV-2 CA’s NTD revealed that its binding mode is highly similar to that of HIV-1 CA (Fig 3B). Most of the residues that form hydrogen bonds and nonbonded contacts in HIV-1 [83] were found to be identical in the structurally equivalent positions of HIV-2 CA [82] (Fig 3C). The highly comparable interaction networks show correlation with the similar efficacy of LEN against HIV-1 and HIV-2 CA.

## 3. Mutations in CA and their effect

Mutations in the HIV-1 CA protein significantly alter the CA’s stability, impacting critical stages of the viral life-cycle, such as reverse transcription and nuclear entry, therefore, altering viral infectivity [84] (Table 1). Several substitutions described in the literature are primarily related to fitness as they disrupt intrinsic CA architecture rather than confer drug resistance, for example, as one of the commonly studied residue mutations, P38A that is located within the central region of helix α2 that forms an 18-helix barrel at the core of the hexamer along with helices α1 and α3. This P38A mutation affects interacting residues such as E29, K30, and P34, destabilizing the core by reducing the interface area between CA subunits [84,85], as summarized in Table 1. Similarly, mutations such as R18A/N21A and Q63/Q67A, and R143A located in the NTD; and R143A, K170A, K203A, and Q219A in the CTD also destabilize the CA, many of these mutations were found to disrupt reverse transcription and lower viral replication efficiency [86].

**Table 1:** HIV CA mutations and their known effects on viral replication. The mutations are shown based on literature data [57,84,85,45,88]. Residues without an apostrophe are in the primary subunit of the HIV-1 capsid hexamer. Single and double apostrophes indicate residues in neighboring subunits within or across hexamers as indicated by source.

Mutation	Affected Residues	Capsid Stability Effect	Functional Impact
P38A [57,84]	P38, E29, K30, P34, E35, V36, I37, M39, S41, A42, L20΄, E28΄, T54΄	Destabilizes cores, reduces interface area	Reduced Infectivity
R18A/N21A [45]	R18, N21	Destabilizes cores	Aberrant core morphology, Impaired reverse transcription
Q63A/Q67A [85]	Q63, Q67	Destabilizes/hyperstabilizes cores	Impaired nuclear import, delayed uncoating
E45A [57,84]	E45, S44, G46, E128, K130, R132, Q50΄, D51΄, P1΄, H12΄, R82	Hyperstabilizes cores, increases interface areas	Preserved reverse transcription, delayed uncoating, impaired nuclear entry
P38A/T216I [84]	P38, T216, I216΄΄, Q219΄΄, V221΄΄, I201΄΄, L202΄΄, K203΄΄, A204΄΄	Partially restores stability	Improved assembly and infectivity compared to P38A alone
E45A/R132T [84]	E45, R132	Reduces hyperstability	

The E45A mutation, altering the interaction of nearby residues like S44, G46, and R132, hyperstabilizes the CA by increasing inter-subunit interface, resulting in a rigid structure that resists timely uncoating [84]. While reverse transcription proceeds, delayed uncoating impedes nuclear entry, decreasing infectivity (due to lattice stabilization). Compensatory mutations, such as T216I with P38A or R132T with E45A, can partially restore stability and infectivity by modulating CA interactions [57,84–86].

In contrast, some mutations selected under treatment pressure are more specifically associated with reduced drug susceptibility, although they may also affect CA fitness. These resistance-associated substitutions do not simply overlap with the broader set of structurally disruptive mutations, rather, they represent a distinct subset of mutations that preserve enough CA function to permit replication while weakening binding of the inhibitor. Taken together, CA mutations fall into at least two partially overlapping categories: those that primarily alter CA fitness, and those that emerge under treatment pressure, that selects a narrower set of substitutions that map to the drug-binding pocket, mediating antiviral resistance and often accompanied by substantial replication defects [87].

## 4. Host factor interactions with HIV CA

As previously mentioned, the interaction between host factors and the HIV CA is critical for viral replication and immune evasion, involving a complex interplay of cellular proteins that either facilitate or restrict viral infection. CypA, a host peptidyl-prolyl isomerase of a molecular weight of 18 kDa [89], plays a pivotal role in HIV-1 infection by certain viral strains and in specific target cells, by interacting with the viral CA protein. CypA binds to the proline-rich loop in the N-terminal domain of CA, stabilizing the CA and facilitating key steps such as reverse transcription, nuclear entry, and integration [50,90]. This interaction enhances viral infectivity, with CypA-deficient virions showing reduced DNA synthesis and infectivity [91]. CypA also modulates nuclear entry by coordinating nucleoporin interactions and counteracting restriction factors like TRIM5α and MxB, though its effects vary by cell type [50,92,93]. In their study, Padron et.al. showed that CypA depletion or inhibition with cyclosporin A reduced proviral integration in CypA-expressing cells, independent of its effects on earlier steps or tripartite motif family of proteins TRIM5α [60]. These findings underscore CypA’s multifaceted role in HIV-1 replication, with its CA-binding function critical to multiple stages of the viral life-cycle.

Nucleoporin 153 (Nup153) is a key component of the nuclear pore complex (NPC) and plays a major role in the nuclear import of HIV-1 and other lentiviruses [94]. It functions at the interface between the cytoplasm and nucleus, mediating the translocation of the viral pre-integration complex through the NPC, by way of direct interaction with the HIV-1 CA, facilitating the docking and translocation of the viral core into the nucleus [95]. This interaction is essential for productive infection, as disruption of the Nup153–CA binding impairs nuclear import and reduces HIV-1’s infectivity, highlighting the critical role of Nup153 in coordinating the early post-entry steps of the viral life-cycle [96]. The binding mode of a NUP153 peptide is shown in Fig 1.

Additionally, Cleavage and Polyadenylation Specificity Factor 6 (CPSF6) plays a critical role in determining HIV integration by facilitating nuclear import and influencing the selection of integration sites. Specifically, interactions between CPSF6 and the viral CA enable the virus to evade peripheral heterochromatin, thereby promoting efficient integration into the host genome [97,98]. The binding mode of a CPSF6 peptide is shown in Fig 1.

Conversely, restriction factors such as TRIM5α and Myxovirus resistance proteins (MxB) target the CA to inhibit infection. TRIM5α recognizes the capsid lattice in a species-specific manner, triggering premature uncoating and proteasomal degradation [99], while MxB restricts uncoating and nuclear import by binding to the CA [100,101].

These interactions highlight the delicate balance between host-mediated facilitation and restriction of HIV infection, with the CA serving as a key interface.

## 5. HIV Capsid inhibitors

HIV CA inhibitors (CIs) represent a novel and promising class of antiretroviral agents that specifically target the CA protein, a critical structural component essential for multiple stages of the HIV life-cycle and viral infectivity. Unlike traditional antiretrovirals that inhibit viral enzymes; such as reverse transcriptase, integrase, or protease, CIs disrupt the structural integrity and functional dynamics of the CA by interfering with processes including capsid assembly, stability, disassembly, and uncoating. A major advancement in this class was the development of GS-6207 (lenacapavir, LEN), a long-acting CI with high potency and a favorable resistance profile. The emergence of such inhibitors marks a significant step forward in HIV therapy, particularly for patients with multidrug-resistant viral strains, underscoring the therapeutic potential of targeting viral structural proteins.

### 5.1 Inhibitors targeting the NTD-CTD interface (binding pocket)

CIs targeting the NTD-CTD interface of the HIV CA represent a strategic approach to disrupt the critical inter-domain interactions necessary for CA assembly and stability [102]. Inhibitors that bind at this interface can destabilize the hexameric lattice by preventing proper domain-domain interactions, thereby impairing capsid formation, maturation, and uncoating processes essential for viral replication. By targeting the NTD-CTD interface, these inhibitors exploit a highly conserved and functionally vital region of the CA, reducing the likelihood of resistance development [71]. Compounds that interfere with this interface can thus effectively inhibit multiple stages of the viral life-cycle, offering a promising avenue for the design of next-generation CA-targeting antiretroviral therapies.

#### 5.1.1 Lenacapavir.

**Fig 4:**
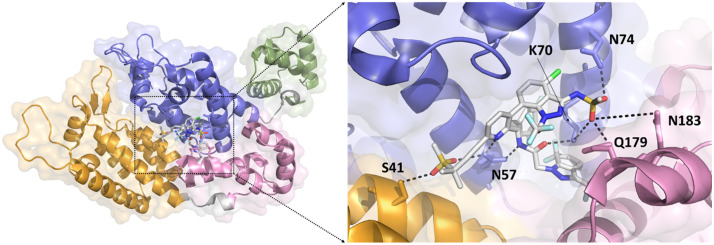
Binding of lenacapavir to HIV-1 CA. Lenacapavir interacting with two CA molecules is represented based on a crystal structure (PDB ID: 6VKV) [70]. Two neighboring monomers are shown, their NTD and CTD domains have blue and green as well as orange and pink colors, respectively. The short inter-domain linkers have gray color. Enlarged view of lenacapavir-binding site is also shown, the key hydrogen-bond interactions are shown by black dashed lines.

Lenacapavir (LEN) (GS-6207), marketed as Sunlenca, is a first-in-class, long-acting inhibitor of the HIV-1 CA. It was approved by the U.S. Food and Drug Administration (FDA) in December 2022 for the treatment of multidrug-resistant (MDR) HIV-1 infection in heavily treatment-experienced adults [103]. This approval was based on data from the Phase 2/3 double-blind, placebo-controlled, global multicenter CAPELLA trial (NCT04150068), in which LEN achieved sustained virologic suppression, with participants reaching an undetectable viral load (<50 copies/mL) by week 52 [104]. Mechanistically, LEN primarily interacts with a hydrophobic pocket that is located between three helices (helix 3, 4, and 5) of NTD in HIV-1 CA monomer and the CTD (helix 8 and 9) of an adjacent monomer within the hexameric assembly. It engages in hydrophobic and electrostatic interactions with the NTD, and forms hydrogen bonds with the CTD, stabilizing the hexamer interface [70]. This interaction results in “locking” the CA in a hyperstable state, disrupting multiple stages in the viral life-cycle that are mediated by the CA [70,105] (Fig 4).

This binding site of LEN can be occupied by other molecules, for example, by small-molecule inhibitor PF-3450074 [71], as well as by host proteins, such as cellular RNA processing factor CPSF6 [72], nuclear import/pore protein NUP153 [73], or protein transport protein Sec24C [74]. Each of these ligands contain a common phenylalanine-glycine (FG) motif that mediates binding to the FG-binding pocket of HIV-1 CA where LEN also binds.

The interaction between LEN and CA monomers is known to depend on key conserved residues in the NTD such as N57, K70 and N74, T54, I73, and N74 in one monomer, and S41 (NTD), Q179 (CTD), and N183 (CTD) in the other [70,83].

*In vitro*, LEN demonstrated an overall excellent half-maximal effective concentration (EC_50_) in the picomolar range of 50–314 pM [70], with EC_50_ of 105 pM against HIV-1 in MT-4 cells, 56 pM in macrophages, and 32 pM in primary CD4+ T cells [106]. In regard to HIV-2, LEN was also shown to inhibit multiple steps of the viral life-cycle with a half-maximal inhibitory concentration (IC_50_) of 206.2 pM against ROD-based HIV-2 [82], and an EC_50_ of 885 pM against two HIV-2 isolates [106].

#### 5.1.2 GS-CA1 and GS-CA2.

GS‑CA1 and its analogue GS-CA2 are highly potent small-molecule inhibitors of the HIV‑1 CA [107,108]. Akin to LEN, they bind with high affinity to a conserved hydrophobic pocket located at the interface of two adjacent CA monomers within the hexameric assembly. Structural studies, including X-ray crystallography and molecular modeling, have revealed extensive van der Waals and hydrogen-bond interactions with key conserved residues such as L56, N57, M66, Q67, K70, N74, and T107 [107]. *In vitro*, the mean EC_50_ of GS-CA1 was shown to be 240 pM in MT-4 cells [107], and around 140 pM in primary human peripheral blood mononuclear cells [109]. GS-CA1 also exhibited efficacy against SIV and HIV-2 *in vitro*, albeit its potency was somewhat lower compared to its effect on HIV-1 [107]. Preclinical models in a rhesus macaques showed that a single 300 mg/kg dose of GS-CA1 reduced the risk of infection by SIV by 97% for 24 weeks [110], with favorable pharmacokinetics and sustained drug levels, supporting its potential as a long-acting therapeutic or prophylactic agent against HIV [107].

#### 5.1.3 BI-1 and BI-2.

BI-1 and its structural analog BI-2 are small-molecule pyrrolopyrazolones that target the NTD of CA protein, stabilizing CA assemblies and preventing uncoating of viral CAs *in vitro*, in addition to competitively preventing the binding of the host factor CPSF6 to the CA NTD-CTD interface, a crucial interaction for nuclear trafficking and integration of the viral genome [111,72]. Structural studies indicate that BI-2 interacts with the binding pocket formed by residues from helices 3, 4, and 5, with residues N57, K70, A105, and T107 playing a vital role in mediating the interaction [111].

In single and multicycle viral replication assays performed in SupT1 and C8166 cells, respectively, BI-1, showed EC_50_ values of 7.5-8.2 μM, while BI-2 demonstrated higher potency with EC_50_ of 1.4-1.8 μM against VSV-G pseudotyped HIV-1. However, the inhibitory effect of these small-molecule inhibitors was only apparent in the early phase of infection, as the molecules failed to inhibit the production of infectious virions [111].

The pharmacokinetics and pharmacodynamics of these inhibitors remain poorly characterized. However, given their relatively high EC_50_ values, further investigation into their properties appears to be of limited utility, particularly in light of the availability of more efficacious alternatives.

#### 5.1.4 CAP-1.

CAP compounds (CAP-1 and CAP-2) are small molecular inhibitors that also belong to this subgroup, binding at the base of the NTD, at the junction of α-helices 1, 2, 3, 4, and 7 [112]. This binding is thought to disrupt the formation of the interphase between one CA NTD and another’s CTD [113]. While CAP-2 was highly cytotoxic, in U1 cell infectivity model, CAP-1 reduced infectivity of HIV-1 by 95% at 100 μM concentration, inhibiting the late phase of the viral life-cycle with no effect on the early phase [112].

**Fig 5:**
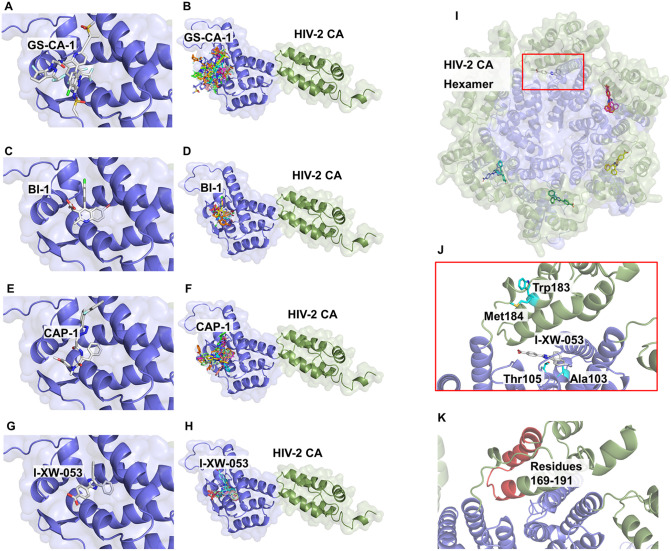
Structure of inhibitors bound to HIV-2 CA. Predicted binding mode of GS-CA-1 **(A)**, BI-1 **(C)**, CAP1 **(E)**, and I-XW-053 **(G)** to HIV-2 CA, the conformers predicted with the highest confidence value are represented for each CI with stick representation mode. All of the ten predicted conformers are also shown for GS-CA-1 **(B)**, BI-1 **(D)**, CAP1 **(F)** with HIV-2 CA monomer, and I-XW-053 **(H)**—taken from hexamer structure, after alignment. **(I)** Structure of I-XW-053 inhibitor-bound to complete HIV-2 CA hexamer structure, the ten conformers are represented as sticks. **(J)** Closeup of I-XW-053 conformer predicted with highest confidence with residues involved in NTD-NTD interface, represented as sticks. **(K)** Residues of the dimerization interface, according to HIV-1 CA numbering, are colored red. The NTD and CTD domains have blue and green colors, respectively, consistently. The HIV-2 CA hexamer structure was modeled with AlphaFold3 [116]. Docking was carried out with DiffDock [115].

Given the scarcity of data, in the frame of this work, we performed modeling and docking simulations to investigate how these CIs interact with the HIV-2 CA. Our results suggest that GS-CA1, BI-1, and CAP-1 also bind in a similar mode as previously described for HIV-1; however, more rigorous computational analyses and detailed binding site characterization will be essential to fully elucidate these interactions, particularly in light of polymorphisms among different viral groups and circulating recombinant forms. Results of our docking experiments are shown in Fig 5.

Moreover, we utilized Ligplot+ [114] to analyze the interactions between HIV-2 CA and the GS-CA-1, BI-1, CAP-1, and I-XW-053 inhibitors, based on the most probable docked conformer derived with DiffDock [115]. Results show that GS-CA-1, and BI-1 were the only ones forming a single hydrogen bond with Asn56 residues. All analyzed inhibitors exhibited extensive nonbonded interactions, indicating stable complex formation. Based on our analysis, the interaction pattern is highly similar, and Asn56, Arg69 as well as Asn73 residues of HIV-2 CA contribute to the binding of each inhibitor (Table 2).

**Table 2:** Interactions between HIV-2 capsid protein and inhibitors. Interacting residues were determined with LigPlot+ [114], based on the most probable docked conformers obtained from DiffDock analysis. The hydrogen bonds are side-chain mediated, and all listed residues (with the exception of Gly104) form predominantly side-chain mediated nonbonded interactions as well.

Inhibitor	Hydrogen bonds	Nonbonded interactions
GS-CA-1	Asn56	Asn52, Asn56, Arg69, Asn73
BI-1	Asn56	Asn52, Leu55, Asn56, Ile68, Arg69, Ile72, Asn73, Ala103, Gly104, Thr105, Tyr129
CAP-1	–	Asn52, Leu55, Asn56, Arg69, Ile72, Asn73, Ala103, Thr105, Tyr129
I-XW-053	–	Leu55, Asn56, Ile68, Arg69, Ile72, Asn73, Ala103, Tyr129

#### 5.1.5 BM.

Modified benzodiazepine (BD) and benzimidazole (BM) compounds have demonstrated inhibitory activity against the HIV-1 CA protein, binding to the same pocket as CAP compounds, with some extension into adjacent regions. Key residues involved in the interaction include F32, H62, V24, and V59 [117]. These compounds primarily target the late phase of the viral replication cycle, as evidenced by their limited activity during early infection stages. They exhibit sub-micromolar inhibitory potency against HIV-1, with BD1 displaying high efficacy in multiple-cycle replication assays [117]. To date, no pharmacokinetic or pharmacodynamic studies have been reported for these compounds, and their activity against HIV-2 remains uncharacterized.

### 5.2 Inhibitors targeting the NTD-NTD Interface (CA-CA interactions)

These compounds typically exploit a hydrophobic pocket formed at the six-fold intra-hexameric NTD-NTD interface [54,118], engaging key residues like W184, M185, and A105 in HIV-1 CA, or the corresponding HIV-2 CA residues W183, M184, and A103 (since position 105 in HIV-2 CA is occupied by threonine residue, Fig 5J). Through hydrogen bonds and hydrophobic interactions, these compounds disrupt proper hexamer assembly. By locking the CA in an aberrant conformation or destabilizing assembled CAs, these inhibitors hinder multiple stages of the viral life-cycle [119]. Example of these type of inhibitors is the I-XW-053; a small organic molecule derived from a parent compound CK026, that demonstrated modest efficacy against a broad range of primary HIV-1 strains with IC_50_ of 22.5 μM, through disruption of the uncoating process, although, it did not inhibit the replication of SIV [120].

### 5.3 Inhibitors targeting the hydrophobic groove (CTD-CTD Interface)

#### 5.3.1 Peptide inhibitors.

Capsid assembly inhibitor (CAI) is a peptide that disrupts HIV-1 CA formation by targeting the dimerization interface. It specifically interacts with residues 169–191 (Fig 5K), encompassing helices 8, 9, and 11 of the HIV-1 CA [121]. This interaction leads to the formation of nonfunctional CA dimers that either lack a critical assembly interface or are incapable of being incorporated into the viral particle [121].

To harness its therapeutic potential, hydrocarbon stapling was employed to convert CAI into a cell-permeable peptide, designated NYAD-1. This modified peptide effectively inhibits both early- and late stages of HIV-1 infection, and demonstrates antiviral activity against a range of HIV-1 strains, with IC_50_ values between 4 and 21 μM [122]. Its analoge NYAD-13 is highly soluble, with a C-terminal proline replaced by three lysine residues. It has demonstrated efficacy with similar IC_50_ ranges, albeit, the cytotoxicity was more pronounced compared to NYAD-1 [122]. Similar peptides NYAD-36, NYAD-66, and NYAD-67 were also effective in inhibiting infection by multiple HIV-1 isolates in PBMC’s with IC_50_ in the micromolar range, with NYAD-67 being the most effective. Additionally, these inhibitors unexpectedly showed dual activity by also binding to the HIV-1 envelope glycoprotein gp120, particularly the V3 loop, inhibiting viral entry in addition to impairing Gag processing [123–125].

CAC1, an N-terminal-acetylated and C-terminal-amidated peptide comprising residues 175–194 of the wild-type HIV-1 CA protein, was designed to mimic the dimerization interface and disrupt CA CTD-CTD interactions [126]. On its own, it showed poor inhibitory activity on HIV-1 production e*x vivo*, with concentrations in the millimolar range being required, however, in combination with a chariot and CAC1-derived peptides, that were created to increase peptide solubility and helical propensity, HIV-1 strain HXB2 production was decreased by roughly an 80% in U87-CD4-CXCR4 cells, albeit, at a high micromolar concentration [127].

Given their nature, peptide inhibitors face significant hurdles for clinical translation, including poor *in vivo* stability, limited cell permeability, and to a major extend low solubility, which have restricted studies to *in vitro* settings. To our knowledge, no human clinical trials or pharmacokinetic studies have been reported for any NYAD peptides to date.

### 5.4 nonspecific inhibitors

PF-3450074 (PF74) is a small-molecule inhibitor that targets the HIV-1 CA by binding to a pocket at the NTD-CTD interface, involving NTD helices 3, 4, 5, and 7, at the junction of two adjacent monomers within the assembled CA hexamer. It forms key interactions with NTD residues N57, M66, Q63, K70 and some CTD residues such as K182, overlapping with the binding sites of host factors CPSF6 and NUP153 [71]. Its binding site is distinct from those of CAP-1 and CAI/NYAD-1 [71]. PF74 disrupts the higher-order structure of the CA hexamer by weakening NTD-CTD interactions, leading to premature uncoating in the early phase of infection, which impairs reverse transcription, nuclear entry, and integration, and weakly inhibits CA assembly in the late phase [71,45]. This compound exhibits antiviral activity with an EC₅₀ of 0.57 μM in MT-2 cell-based assays against the HIV-1 NL4-3 strain. However, its clinical development is constrained by modest potency in the micromolar range, limited metabolic stability, and the rapid emergence of resistance mutations.

Ebselen, an organoselenium compound with established anti-inflammatory, antimicrobial, and cytoprotective properties [128,129], was identified as a CA-targeting agent through a time-resolved fluorescence resonance energy transfer (TR-FRET) high-throughput screening assay. It was shown to directly bind the CTD of the HIV-1 CA and inhibit early-stage replication events of the HIV-1 NL4-3 isolate in HeLa-CD4-LTR-β-galactosidase (LacZ) reporter cells, with an EC₅₀ of ~2 μM [130]. This molecule was shown to covalently bind the highly conserved cysteine residues (Cys198, Cys218) via a selenylsulfide linkage in the CA-CTD, increasing the stability of the CA and impairing uncoating [130]. However, its nonspecific reactivity with cysteine-containing proteins, including HIV-1 and host proteins, raises concerns about toxicity and off-target effects, although, a particular benefit was shown when ebselen exhibited a moderate inhibition of LEDGF/p75-IN interaction, an inhibition that was reversed by dithiothreitol (DTT) [131]. Ebselen was also found to induce oxidative stress via redox cycling [132]. The lack of selectivity and unknwon pharmacokinetic profile, most likely have precluded clinical development to combat HIV-1, limiting its use to preclinical studies.

## 6. New inhibitors and perspectives

More recently, compound H27; a novel small-molecule inhibitor was found to interfere with HIV-1 CA without altering assembly or uncoating. This inhibitor was found to exert its effect primarily by specifically disrupting proper interaction with the nuclear import machinery of the PIC, ultimately hindering nuclear entry [133]. While the exact binding site of this inhibitor to HIV-1 CA has not yet been established, E45L and G46 were found to be critical residues for the inhibitor’s action [133]. The inhibition of HIV-1 replication was dose-dependent, with IC_50_ values of 2.9–5.6 µM in primary lymphocytes, using pseudotyped, NL4-3, and BaL HIV-1 isolates [133]. Its specificity and lack of cross-resistance with PF74 and lenacapavir suggest H27 as a promising candidate for further development in combination antiretroviral therapies, though preclinical pharmacokinetic and toxicity studies are needed.

Designed as a robust alternative to conventional antibodies, Designed Ankyrin Repeat Proteins (DARPins) retain the high affinity and specificity for target binding characteristic of antibodies, while offering distinct advantages in terms of physicochemical stability and cost-effective production [134,135]. Utilizing highly diverse DARPin DNA libraries, potent inhibitors and target-specific binding proteins across a broad range of biological systems were identified.

Targeting HIV, DARPins were designed to interact with CD4 receptors present on the surface of HIV target cells, competing with gp120 for binding and thereby inhibiting viral entry and subsequent infection in low nanomolar range [136].

A phage-displayed ankyrin-repeat protein library was screened against the MA-CA domain of HIV-1 Gag, identifying AnkGAG1D4, which binds the NTD of the CA protein [137]. Stable expression of AnkGAG1D4 in SupT1 cells reduced HIV-1 NL4-–3 infectivity by interfering with late-stage processes, including viral assembly and budding. N-myristoylation of AnkGAG1D4 enhanced its antiviral activity by targeting it to the plasma membrane, further impairing viral budding. This effect likely results from sequestration of Gag proteins and/or competition for membrane anchoring sites essential for virion formation. Additionally, AnkGAG1D4 may interfere with CypA incorporation into virions, though this effect requires further investigation [137].

S45Y substitution significantly enhanced the binding affinity of AnkGAG1D4 for the monomeric HIV-1 CA [138], and a dimeric form connected by a (G_4_S)_4_ linker was developed to further improve its affinity through increased flexibility and solubility [139]. Despite its promising *in vitro* efficacy, AnkGAG1D4’s intracellular delivery challenges and lack of clinical data limit its therapeutic potential.

GSK878 is a recently developed HIV-1 inhibitor that targets the mature CA hexamer, binding to a pocket similar to that of the established CA inhibitor PF-74 [140]. It was shown to exert potent antiviral activity against HIV-1 reporter virus in MT-2 cells, with mean EC_50_ in the low picomolar range [140]. This inhibitor alters the stability of the CA core, impairing nuclear import and proviral integration, and while it remains to be proven, GSK878 is also thought to weakly inhibit later stages of the viral life-cycle, perhaps by binding to the CA domain of Gag, through interactions with the NTD of CA region [140]. Sharing a similar pocket with PF-74, mutations such as L56I, M66I, Q67H, N74D, T107N, and the Q67H/N74D combination were found to decrease susceptibility to GSK878, with M66I, Q67H/N74D, and L56I exerting the strongest effects on antiviral activity [140].

VH4004280 (VH‑280) is a novel, orally administered HIV‑1 capsid inhibitor that binds to a conserved pocket within the mature capsid hexamer targeting the CPSF6/nucleoporin binding pocket and disrupts both early and late steps of the viral life-cycle, with half‑maximal effective concentrations in the picomolar range against a broad panel of HIV‑1 laboratory strains and clinical isolates [141]*. In vitro* analysis revealed that Q67H and adjacent substitutions such as A105E and T107D/N were key resistance‑associated changes [142]. In a phase 1 study in HIV negative adults, VH‑280 exhibited a long oral half‑life of ~6–9 days, and a favorable safety and drug‑interaction profile, supporting its further development as a long‑acting component of HIV‑1 treatment and prevention regimens [141].

A related capsid inhibitor, VH4011499 (VH‑499), with a comparable antiviral potency and resistance profile, is currently under clinical development as both an oral and long‑acting antiretroviral [142]. In a short‑term phase 2a proof‑of‑concept trial in treatment‑naive individuals with HIV‑1, higher doses of oral VH‑499 led to substantial reductions in viral load, whereas lower systemic exposure permitted the emergence of resistance‑associated CA amino acid substitutions, emphasizing the need for adequate drug levels and optimized dosing strategies (ClinicalTrials.gov ID NCT06039579). Ongoing phase 1 studies are further exploring long‑acting injectable formulations of VH‑499 (ClinicalTrials.gov ID NCT06012136). To facilitate comparative analysis, we prepared a comprehensive summary of currently reported CIs and their antiviral efficacy against HIV-1, and, where available, HIV-2. The relevant data have been systematically compiled and presented in Table 3.

**Table 3:** Summary of reported HIV capsid inhibitors and their antiviral efficacy against HIV-1 and, where available, HIV-2. The table includes representative efficacy values, experimental cell types, and viral model used in the respective studies.

Inhibitor	Reported efficacy	Cell type	Virus
LEN [70,106]	EC_50_: 32–314 pM	MT-4, macrophages, CD4+ T-cells	HIV-1
LEN [82]	IC_50_: 206.2 pM	Jurkat cells	HIV-2 ROD
LEN [106]	EC_50_: 885 pM	CD4+ T-cells, macrophages	HIV-2 Isolates
GS-CA1 [107]	Mean EC_50_: 240 pM	MT-4 cells	HIV-1
GS-CA1 [109]	EC_50_: ~140 pM	Primary PBMCs	HIV-1
GS-CA1 [107]	EC_50_ > 1 nM	Primary PBMCs	SIV/HIV-2
BI-1 [111]	EC_50_: 7.5-8.2 μM	SupT1/C8166 cells	HIV-1 (VSV-G pseudotyped)
BI-2 [111]	EC_50_: 1.4-1.8 μM	SupT1/C8166 cells	HIV-1 (VSV-G pseudotyped)
CAP-1 [112]	95% infectivity reduction at 100 μM	U1 cell infectivity model	HIV-1
BD1 [117]	Sub-micromolar potency	C8166-LTR-luciferase cells	HIV-1
I-XW-053 [120]	IC_50_: 22.5 μM	Cf2Th-CCR5 cells	HIV-1
NYAD-1 [122]	IC_50_: 4–21 μM	MT-2/PBMCs	HIV-1
NYAD-36/66/67 [122,123]	IC_50_: low micromolar range	MT-2	HIV-1
CAC1 derivatives [127]	~80% inhibition at high μM	U87-CD4-CXCR4 cells	HIV-1
PF74 [71]	EC_50_: 0.57 μM	MT-2 cellS	HIV-1
Ebselen [130]	EC_50_: ~2 μM	HeLa-CD4-LTR-β-gal (NL4-3, early replication)	HIV-1
H27 [133]	IC_50_: 2.9–5.6 μM	Primary lymphocytes (pseudotyped/NL4-3/BaL)	HIV-1
GSK878 [140]	Mean EC50: low picomolar	MT-2 cells	HIV-1
VH-280/ VH-499 [141]	EC_50_: picomolar range	MT-2 cells (NL4-3-derivative)	HIV-1

## 7. CA mutations and mechanisms of resistance development

Resistance mutations in the HIV-1 CA present significant challenges to the efficacy and clinical development of CA-targeting inhibitors. The mutations primarily cluster within or adjacent to the drug-binding pocket, reducing inhibitor affinity while often imposing significant replication fitness costs.

For example, LEN is associated with a spectrum of resistance mutations of HIV-1 CA, including L56I, M66I, Q67H, T107N, K70N, N74D/S, and A105E [143,144]. These mutations impose substantial replication fitness costs at least in the case of HIV-1, primarily through structural reorganization of the CA lattice that disrupts the precise spatiotemporal dynamics required for uncoating, reverse transcription, and nuclear import [87,145]. Clinical isolates from CAPELLA and CALIBRATE trials carrying primary resistance mutations such as M66I exhibited replication capacities as low as 13–17% of wild-type virus, with phenotypic analyses confirming 4- to >800-fold reductions in susceptibility to LEN, accompanied by marked uncoating delays and Gag-Pro processing defects [87]. Combinatorial mutations, such as Q67H/T107N and Q67H/N74D, further amplify resistance levels by more than 60-fold and 1,000-fold, respectively [83,146].

GS-CA1, a compound structurally related to LEN, shares a similar resistance profile, with overlapping mutations of HIV-1 CA including L56I, N57S, M66I, Q67H, Q67Y, and N74D. These mutations result in significant reductions in inhibitor potency [107]. Although individual mutations such as K70R and T107N do not independently confer substantial resistance, their presence in combination with Q67H leads to over a 50-fold increase in EC_50_ values [107]. While GS-CA2, a structural analogue of GS-CA1, is presumed to exhibit a similar resistance profile, specific data on resistance mutations remain limited.

Modified benzodiazepine (BD) and benzimidazole (BM) inhibitors are influenced by distinct sets of resistance-associated mutations. For BD inhibitors, *in vitro* data; although limited, indicate selection of mutations of HIV-1 CA such as V36T, G61E, V27A/I, and T58I. In the case of BM compounds, mutations including K30R, S33G, and T58I were identified, all contributing to multi-fold increases in EC_50_. Among these, V36T exerted the most pronounced effect on BD compound activity, whereas T58I most severely impaired BM compound efficacy. Notably, the V36T/G208R combination significantly diminished BD inhibitor potency, while the K30R/G208R combination had an analogous detrimental effect on BM inhibitors [117].

Resistance to PF74 is conferred by substitutions in HIV-1 CA, such as Q67H, S41A, V165I, and L172I. High-level resistance is observed with combinations including S41A/Q67H and either V165I or L172I [147]. Less-characterized inhibitors like BI-1 and BI-2, which bind to the same site in the NTD as PF74 [88,111], are likely affected by resistance mutations including A105S, T107N, N57A, and N57S, which may alter the binding affinity [148].

Unlike some LEN resistance mutations (M66I), which can severely impair viral replication capacity, these molecules tend to select resistance pathways involving compensatory mutations that preserve greater viral fitness [149].

CAP-1 and CAP-2 interact with a unique hydrophobic pocket in the NTD near the linker region in HIV-1 CA, inducing conformational changes [148]. However, resistance profiles for these compounds have not yet been characterized. Docking studies suggest that I-XW-053 depends critically on residues I37 in helix 2; and R173 in helix 8 for effective binding [120]

For the peptide inhibitor CAI, effective binding requires structural integrity of the HIV-1 CA CTD dimer interface, particularly helix 9. Mutations within the 169–191 region can confer resistance by disrupting this interface, with W184A and M185A mutations markedly reducing CAI binding affinity [121].

The cell-penetrating derivatives of CAI (the NYAD family of peptides) also target the HIV-1 CA CTD’s hydrophobic groove and are likely impacted by similar resistance mutations. Interestingly, treatment with NYAD-36 led to the emergence of mutations V120Q (in the C1 region) and A327P (at the base of the V3 loop) in the envelope glycoprotein gp120, which conferred substantial resistance in cell culture assays [123].

CAC1 and its derivative peptides predominantly bind to the same region of the CA-CTD, particularly helix 9, interacting with residues 184, 185, and 188, as well as residues 150, 154, 190, 200, and 203 near the CTD dimerization interface [127]. A well-defined resistance mutation profile for CAC1 remains to be established.

Regarding the recently identified small-molecule inhibitor H27, which targets CA-mediated nuclear import, two nonpolymorphic mutations, E45L and G46A, have been shown to confer resistance. These hyperstabilizing mutations are likely to stabilize the CA to resist H27’s disruption [45,133].

Ebselen, a covalent inhibitor targeting cysteine residues Cys198 and Cys218, has not yet been associated with any reported resistance mutations. Likewise, no resistance mutations have been identified against Designed Ankyrin Repeat Proteins (DARPins) that target the viral CA.

Table 4 summarizes the inhibitors and the resistance‑associated mutations reported thus far.

**Table 4:** CA inhibitors and their associated resistance mutations.

Inhibitor	Stage	Known Resistance Mutations in HIV-1 CA	Notes
Lenacapavir	Approved and in clinical use (treatment and PrEP)	L56I, M66I, Q67H, T107N, K70N, N74D/S, A105E	Significant reduction in efficacy, Q67H/T107N and Q67H/N74D combinations increase resistance >60-fold and >1000-fold
GS-CA1	*In vitro*/experimental (preclinical lead)	L56I, N57S, M66I, Q67H, Q67Y, N74D	Overlapping resistance with LEN, notable increase in resistance
GS-CA2	*In vitro*/experimental	Presumed similar to GS-CA1, specific mutations not detailed	Limited data, but likely comparable resistance profile
BD inhibitors	*In vitro*/experimental	V36T, G61E, V27A/I, T58I, V36T/G208R (combination)	V36T reduces BD activity significantly, V36T/G208R combination markedly reduces BD efficacy
BM inhibitors	*In vitro*/experimental	K30R, S33G, T58I, K30R/G208R (combination)	T58I most detrimental to BM efficacy, K30R/G208R combination severely reduces BM activity
PF74	*In vitro*/experimental	Q67H, S41A, V165I, L172I, S41A/Q67H + V165I or L172I	High-level resistance conferred by combination mutations
BI-1/ BI-2	*In vitro*/experimental	A105S, T107N, N57A, N57S	Likely to affect binding affinity
CAP-1/ CAP-2	*In vitro*/experimental	Not characterized	Resistance profile unknown
I-XW-053	*In vitro*/experimental	I37, R173 based on docking dependence	Binding highly dependent on these residues, resistance mutations not confirmed but predicted
CAI	*In vitro*/experimental	W184A, M185A, region 169–191	Mutations disrupt CTD dimerization interface, significantly reduce binding affinity
NYAD	*In vitro*/experimental	V120Q, A327P (V3 loop of gp120)	Substantial resistance in cell culture; mutations outside CA domain
CAC1	*In vitro*/experimental	Residues W184, M185, T188, D150, R154, L190, I200, K203 (predicted interaction sites)	Resistance mutation profile not yet defined
H27	*In vitro*/experimental	E45L, G46A	Hyperstabilizing mutations, likely disrupt CA integrity and confer resistance
Ebselen	*In vitro*/experimental – repurposed	None reported	No known resistance mutations
DARPins	*In vitro*/experimental	None reported	No known resistance mutations
VH4004280	Investigational (Phase I–II)	Q67H, A105E T107D/N	–
VH‑4011499	Investigational (Phase I–II)	Q67H, A105E T107D/N	–

## 8. Major Clinical trials assessing the efficacy of LEN-based combinations

There is increasing scientific evidence on the clinical applicability of LEN. Following the promising approval studies, more real-world data are accumulating on the most appropriate clinical scenarios in which LEN might be used. The phase 2/3 CAPELLA trial evaluated LEN in heavily treatment-experienced adults with multidrug-resistant HIV. Patients received various regimens, including LEN monotherapy or in combination with background ART [104]. Primary endpoint (a decrease of > 0.5 log10 copies per milliliter in the viral load by day 15) was achieved in 88% of patients receiving LEN vs 17% in the placebo group with optimized background therapy. In this trial, safety profile was acceptable, with injection-site reaction being the most common adverse event (63%). With regards to resistance, LEN-associated CA mutations developed in eight patients, however, half of these patients experienced re-suppression during LEN administration. Notably, the 3-year results were impressive in terms of efficacy, and maintaining viral suppression in 85% of the patients, with the exclusion of missing data. No new resistance was detected after week 104, and the frequency of injection-site reactions declined over time [150].

The CALIBRATE study was a phase 2 randomized study in treatment naïve HIV positive individuals. LEN was co-administered with NRTI backbone evaluating four arms: Arm 1 received LEN subcutaneously every 26 weeks (after oral loading) with oral daily emtricitabine (F)/tenofovir alafenamide (TAF) followed by TAF + LEN or bictegravir (BIC) + LEN (Arm 2). Arm 3 received oral LEN with F/TAF, while Arm 4 recieved BIC/F/TAF. All combinations provided virological suppression at week 54, between 85% and 92% [151]. Apart from these published hall-mark trials, there are currently 24 studies registered at the clinicaltrials.gov platform, with 3 completed, 12 active not recruiting, and 9 studies awaiting participants [152]. Among these, the ARTISTRY-1 study (NCT05502341) is noteworthy, which evaluates the efficacy and tolerability of switching from a stable baseline regimen to an investigational once-daily fixed-dose combination of LEN and bictegravir. Furthermore, studies are ongoing with the combination of the novel nucleoside reverse transcriptase translocation inhibitor islatravir and LEN, administered once weekly, in virologically suppressed individuals (NCT05052996, NCT06630286, and NCT06630286). The combination of the broadly neutralizing antibodies teropavimab and zinlirvimab with LEN appears to be a paradigm-shifting approach, currently under evaluation in virologically suppressed adults, with the potential for biannual dosing (NCT05729568, NCT04811040) [152,153].

Importantly, beyond its role in treatment, LEN has proven to be an efficient option for pre-exposure prophylaxis (PrEP) in the PURPOSE trials, owing to its long half-life, infrequent administration, and good tolerability [154,155]. While there are still ongoing PURPOSE trials (NCT06101342, NCT06101329, NCT06513312), the reassuring safety and efficacy results in diverse patient populations led to the recent approval of LEN in June 2025 as PrEP, administered subcutaneously twice a year. This long-acting formulation may help overcome key barriers to prevention, such as the daily pill burden or frequent clinic visits. A potential game changer in prevention could be the ongoing once-yearly LEN PrEP study (NCT07047716).

## 9. Lenacapavir after the dolutegravir era: promise and pitfalls

The development of LEN bears a notable parallel to the emergence of DTG, as both agents were landmarks in ART, initially generating enthusiasm for their potential use in monotherapy and salvage settings, alongside their integration into cART. INSIs such as DTG established themselves as highly potent inhibitors with high barrier to resistance, though resistance can still arise under selective pressure, particularly with suboptimal adherence [156]. Early clinical evidence indicates that LEN is similarly potent, with treatment‑emergent mutations associated with reduced drug susceptibility, yet often accompanied by marked impairments in viral replication fitness [87]. Early reports emphasize that LEN–selected resistance typically emerges in the context of incomplete viral suppression or monotherapy-like conditions, reinforcing the need to reserve the drug for cART rather than standalone use [157]. In this context, optimism about LEN is justified by its unique mechanism and high‑barrier profile, but the experience with DTG and the emerging resistance data caution that long‑term success is not guaranteed, and will be highly dependent on careful regimen design and adherence.

## 10. Conclusion and future perspectives

CAIs are increasingly being considered in the context of HIV cure strategies, because their activity at both early and late stages of the viral life-cycle may help suppress low‑level or intermittent replication, and reduce the seeding or expansion of latent reservoirs. Their long half‑lives, high genetic barriers to resistance, and targeting of highly conserved CA pockets make these agents attractive components of intensified regimens during treatment‑pause studies or latency‑modulation approaches [158]. In this framework, drugs such as LEN and related CAIs could complement broadly neutralizing antibodies, immune‑modulating agents, and latency‑reversing strategies within future HIV‑cure‑oriented combinations, although their precise impact on persistent reservoirs and post‑treatment control remains unclear.

Despite the enthusiasm surrounding the clinical approval of LEN, significant challenges remain. While this drug represents a therapeutic milestone as the first in class targeting the CA to gain FDA approval, many other potential inhibitors are under evaluation, but remain in preclinical phases, marred by pharmacokinetic hurdles, poor solubility or modest efficacy. LEN’s success is partly due to its favorable pharmacokinetics, but replicating this profile is chemically challenging.

Several concerns merit critical discussion. A major question is the clinical relevance of HIV-1 CA (and more importantly HIV-2 CA) polymorphisms across groups, and their impact on CI susceptibility. As described previously, LEN binds to a highly conserved pocket at the interface between CA monomers, but point mutations have already been shown *in vitro* to confer resistance or reduce susceptibility. These mutations can arise under antiretroviral pressure, but importantly, some CA variants bearing similar changes may exist naturally, particularly in clade B and circulating recombinant forms (CRF’s). Compounding this issue is the relative lack of comprehensive global surveillance for CA mutations in clinical isolates, unlike integrase or reverse transcriptase inhibitors, where genotypic resistance testing is standard, no such infrastructure exists yet for CA-targetting drugs, giving their recent debut. Therefore, an early massive deployment of LEN in diverse global HIV infected populations without robust combination strategies, surveillance for emerging resistance mutations, and attention to adherence and pharmacokinetic “tailing” could result in unpredictable efficacy, and potentially contradict favorable preclinical results. It could also undermine treatment programs by contributing to breakthrough infections and the selection of resistant variants, even if *in‑vitro* data and clinical‑trial efficacy appear highly favorable. In the context of PrEP, some have argued, and reasonably so, that CA‑targeting antivirals should be restricted to compounds that act at early stages of infection and bind to sites associated with a higher genetic barrier to resistance, while avoiding maturation‑specific inhibitors or regions prone to polymorphisms [159]. An underexplored concern is whether long-term use of other antiretroviral classes may induce selective pressure that indirectly affect CA structure or function. In such case, compensatory mutations elsewhere in the viral genome could result in alteration to the CA structure, potentially altering binding affinity or inhibitor access. This is indeed relevant given LENs long half-life.

Finally, in countries with low socioeconomic status and lower income; where HIV burden is highest, the cost-effectiveness of CIs remains uncertain, particularly when first line regimens are available as generics. Indeed, the very features that make long-acting formulations an attractive choice; such as reduced dosing frequency, are also those that may be inaccessible in resource-limited settings, especially given the current political climate [160–162].

In conclusion, while CA inhibitors mark a novel frontier in antiretroviral therapy, their implementation must proceed with caution and rigorous surveillance, at least for the short term, especially when mono-and dual antiretroviral therapy protocols are on the horizon.

## References

[1] World Health Organization. Hiv data and statistics. Available from: https://www.who.int/teams/global-hiv-hepatitis-and-stis-programmes/hiv/strategic-information/hiv-data-and-statistics. Accessed 2025 August 19.

[2] Collaborators GH. Global, regional, and national burden of hiv/aids, 1990-2021, and forecasts to 2050, for 204 countries and territories: The global burden of disease study 2021. The Lancet HIV. 2024;11:e807–22. 10.1016/S2352-3018(24)00212-1 PMC11612058 39608393

[3] Coffin JM, Hughes SH, Varmus H. Retroviruses. Plainview, N.Y.: Cold Spring Harbor Laboratory Press; 1997. 21433340

[4] Nyamweya S, Hegedus A, Jaye A, Rowland-Jones S, Flanagan KL, Macallan DC. Comparing HIV-1 and HIV-2 infection: lessons for viral immunopathogenesis. Rev Med Virol. 2013;23(4):221–40. doi: 10.1002/rmv.1739 23444290

[5] World Health Organization. Hiv statistics, globally and by who region. Available from: https://www.who.int/teams/global-hiv-hepatitis-and-stis-programmes/hiv/strategic-information/hiv-data-and-statistics. 2025. Accessed 2026 May 2.

[6] Campbell-Yesufu OT, Gandhi RT. Update on human immunodeficiency virus (HIV)-2 infection. In: Clin Infect Dis. 2011;52:780–7. 21367732 10.1093/cid/ciq248 PMC3106263

[7] Faria NR, Hodges-Mameletzis I, Silva JC, Rodés B, Erasmus S, Paolucci S, et al. Phylogeographical footprint of colonial history in the global dispersal of human immunodeficiency virus type 2 group A. J Gen Virol. 2012;93(Pt 4):889–99. doi: 10.1099/vir.0.038638-0 22190015 PMC3542711

[8] Visseaux B, Damond F, Matheron S, Descamps D, Charpentier C. Hiv-2 molecular epidemiology. Infect Genet Evol. 2016;46:233–40. 27530215 10.1016/j.meegid.2016.08.010

[9] Gojobori T, Moriyama EN, Ina Y, Ikeo K, Miura T, Tsujimoto H, et al. Evolutionary origin of human and simian immunodeficiency viruses. Proc Natl Acad Sci U S A. 1990;87(11):4108–11. doi: 10.1073/pnas.87.11.4108 1693430 PMC54056

[10] Chen Z, Telfier P, Gettie A, Reed P, Zhang L, Ho DD, et al. Genetic characterization of new West African simian immunodeficiency virus SIVsm: geographic clustering of household-derived SIV strains with human immunodeficiency virus type 2 subtypes and genetically diverse viruses from a single feral sooty mangabey troop. J Virol. 1996;70(6):3617–27. doi: 10.1128/JVI.70.6.3617-3627.1996 8648696 PMC190237

[11] Clavel F, Guyader M, Guétard D, Sallé M, Montagnier L, Alizon M. Molecular cloning and polymorphism of the human immune deficiency virus type 2. Nature. 1986;324(6098):691–5. doi: 10.1038/324691a0 3025743

[12] Barré-Sinoussi F, Chermann JC, Rey F, Nugeyre MT, Chamaret S, Gruest J, et al. Isolation of a T-lymphotropic retrovirus from a patient at risk for acquired immune deficiency syndrome (AIDS). Science. 1983;220(4599):868–71. doi: 10.1126/science.6189183 6189183

[13] Bock PJ, Markovitz DM. Infection with HIV-2. AIDS. 2001;15 Suppl 5:S35-45. doi: 10.1097/00002030-200100005-00006 11816173

[14] Esbjornsson J, Mansson F, Kvist A, da Silva ZJ, Andersson S, Fenyo EM, et al. Long-term follow-up of hiv-2-related aids and mortality in guinea-bissau: a prospective open cohort study. The Lancet HIV. 2018. 10.1016/S2352-3018(18)30254-6 30392769

[15] Marchant D, Neil SJD, McKnight Á. Human immunodeficiency virus types 1 and 2 have different replication kinetics in human primary macrophage culture. J Gen Virol. 2006;87(Pt 2):411–8. doi: 10.1099/vir.0.81391-0 16432029

[16] Calado M, Matoso P, Santos-Costa Q, Espirito-Santo M, Machado J, Rosado L, et al. Coreceptor usage by HIV-1 and HIV-2 primary isolates: the relevance of CCR8 chemokine receptor as an alternative coreceptor. Virology. 2010;408(2):174–82. doi: 10.1016/j.virol.2010.09.020 20947116

[17] McKnight A, Dittmar MT, Moniz-Periera J, Ariyoshi K, Reeves JD, Hibbitts S, et al. A broad range of chemokine receptors are used by primary isolates of human immunodeficiency virus type 2 as coreceptors with CD4. J Virol. 1998;72(5):4065–71. doi: 10.1128/JVI.72.5.4065-4071.1998 9557695 PMC109635

[18] Fenrick R, Malim MH, Hauber J, Le SY, Maizel J, Cullen BR. Functional analysis of the Tat trans activator of human immunodeficiency virus type 2. J Virol. 1989;63(12):5006–12. doi: 10.1128/JVI.63.12.5006-5012.1989 2555537 PMC251160

[19] Esbjörnsson J, Månsson F, Kvist A, Isberg P-E, Biague AJ, da Silva ZJ, et al. Increased survival among HIV-1 and HIV-2 dual-infected individuals compared to HIV-1 single-infected individuals. AIDS. 2014;28(7):949–57. 24812673

[20] Mahdi M, Szojka Z, Mótyán JA, Tőzsér J. Inhibitory effects of HIV-2 Vpx on replication of HIV-1. J Virol. 2018;92(14):e00554-18. doi: 10.1128/JVI.00554-18 29743354 PMC6026746

[21] De Wolf F, Roos M, Lange JM, Houweling JT, Coutinho RA, van der Noordaa J, et al. Decline in CD4+ cell numbers reflects increase in HIV-1 replication. AIDS Res Hum Retroviruses. 1988;4(6):433–40. doi: 10.1089/aid.1988.4.433 2905892

[22] Fahey JL, Taylor JM, Detels R, Hofmann B, Melmed R, Nishanian P, et al. The prognostic value of cellular and serologic markers in infection with human immunodeficiency virus type 1. N Engl J Med. 1990;322(3):166–72. doi: 10.1056/NEJM199001183220305 1967191

[23] World Health Organization. Overview of WHO recommendations on HIV and sexually transmitted infection testing, prevention, treatment, care and service delivery. 2026. Available from: https://www.who.int/publications/i/item/B09471

[24] Magomere E, Olwal CO, Tetteh BE, Appeaning M, Ndung’u T, Kyei GB, et al. The confluence of HIV-1 and HIV-2: implications for disease progression and insights for therapy. Int J Microbiol. 2025;2025:3145677. doi: 10.1155/ijm/3145677 40687432 PMC12271722

[25] Travers K, Mboup S, Marlink R, Guèye-Nidaye A, Siby T, Thior I, et al. Natural protection against HIV-1 infection provided by HIV-2. Science. 1995;268(5217):1612–5. doi: 10.1126/science.7539936 7539936

[26] Pinto LA, Covas MJ, Victorino RM. T-helper cross reactivity to viral recombinant proteins in HIV-2-infected patients. AIDS. 1993;7(10):1389–91. doi: 10.1097/00002030-199310000-00016 8267915

[27] Bertoletti A, Cham F, McAdam S, Rostron T, Rowland-Jones S, Sabally S, et al. Cytotoxic T cells from human immunodeficiency virus type 2-infected patients frequently cross-react with different human immunodeficiency virus type 1 clades. J Virol. 1998;72(3):2439–48. doi: 10.1128/JVI.72.3.2439-2448.1998 9499105 PMC109544

[28] Nuwagaba J, Li JA, Ngo B, Sutton RE. 30 years of HIV therapy: current and future antiviral drug targets. Virology. 2025;603:110362. doi: 10.1016/j.virol.2024.110362 39705895 PMC11788039

[29] Department of Health and Human Services. Guidelines for the use of antiretroviral agents in adults and adolescents with HIV. 2025. Available from: https://clinicalinfo.hiv.gov/en/guidelines/adult-and-adolescent-arv

[30] Eggleton JS, Nagalli S. Highly active antiretroviral therapy (HAART). Statpearls. Treasure Island (FL); 2026. 32119420

[31] Kemnic TR, Patel P, Gulick PG. Hiv antiretroviral therapy. Statpearls. Treasure Island (FL); 2026. 30020680

[32] Scarsi KK, Havens JP, Podany AT, Avedissian SN, Fletcher CV. HIV-1 Integrase inhibitors: a comparative review of efficacy and safety. Drugs. 2020;80(16):1649–76. doi: 10.1007/s40265-020-01379-9 32860583 PMC7572875

[33] Hodges-Mameletzis I, Dalal S, Msimanga-Radebe B, Rodolph M, Baggaley R. Going global: the adoption of the World Health Organization’s enabling recommendation on oral pre-exposure prophylaxis for HIV. Sex Health. 2018;15(6):489–500. doi: 10.1071/SH18125 30496718

[34] Antiretroviral Therapy Cohort Collaboration. Survival of HIV-positive patients starting antiretroviral therapy between 1996 and 2013: a collaborative analysis of cohort studies. Lancet HIV. 2017;4(8):e349–56. doi: 10.1016/S2352-3018(17)30066-8 28501495 PMC5555438

[35] Trickey A, Sabin CA, Burkholder G, Crane H, d’Arminio Monforte A, Egger M, et al. Life expectancy after 2015 of adults with HIV on long-term antiretroviral therapy in Europe and North America: a collaborative analysis of cohort studies. Lancet HIV. 2023;10(5):e295–307. doi: 10.1016/S2352-3018(23)00028-0 36958365 PMC10288029

[36] National Institutes of Health (NIH) Office of AIDS Research (OAR). Fda-approved hiv medicines. Available from: https://hivinfo.nih.gov/understanding-hiv/fact-sheets/fda-approved-hiv-medicines. Accessed 2023 May 12.

[37] Adolescents PoAGfAa. Guidelines for the use of antiretroviral agents in adults and adolescents with HIV. Department of Health and Human Services; 2023.

[38] World Health Organization. Who updated recommendations on hiv clinical management: Recommendations for a public health approach. 2026. 41533830

[39] Shah SS, M MPH, AAHIVS. Diagnosis and management of hiv-2 in adults. Available from: https://www.hivguidelines.org/guideline/hiv-2/?mytab=tab_3&mycollection=hiv-testing-acute-infection/#table-1. Accessed 2026 May 7.

[40] Rossi E, Meuser ME, Cunanan CJ, Cocklin S. Structure, function, and interactions of the HIV-1 capsid protein. Life (Basel). 2021;11(2):100. doi: 10.3390/life11020100 33572761 PMC7910843

[41] Francis AC, Melikyan GB. Single HIV-1 imaging reveals progression of infection through CA-dependent steps of docking at the nuclear pore, uncoating, and nuclear transport. Cell Host Microbe. 2018;23(4):536-548.e6. doi: 10.1016/j.chom.2018.03.009 29649444 PMC5901770

[42] Cosnefroy O, Murray PJ, Bishop KN. HIV-1 capsid uncoating initiates after the first strand transfer of reverse transcription. Retrovirology. 2016;13(1):58. doi: 10.1186/s12977-016-0292-7 27549239 PMC4994286

[43] Freed EO. HIV-1 assembly, release and maturation. Nat Rev Microbiol. 2015;13(8):484–96. doi: 10.1038/nrmicro3490 26119571 PMC6936268

[44] AlBurtamani N, Paul A, Fassati A. The role of capsid in the early steps of HIV-1 infection: new insights into the core of the matter. Viruses. 2021;13(6):1161. doi: 10.3390/v13061161 34204384 PMC8234406

[45] Forshey BM, von Schwedler U, Sundquist WI, Aiken C. Formation of a human immunodeficiency virus type 1 core of optimal stability is crucial for viral replication. J Virol. 2002;76(11):5667–77. doi: 10.1128/jvi.76.11.5667-5677.2002 11991995 PMC137032

[46] Jacques DA, McEwan WA, Hilditch L, Price AJ, Towers GJ, James LC. HIV-1 uses dynamic capsid pores to import nucleotides and fuel encapsidated DNA synthesis. Nature. 2016;536(7616):349–53. doi: 10.1038/nature19098 27509857 PMC4998957

[47] Mallery DL, Márquez CL, McEwan WA, Dickson CF, Jacques DA, Anandapadamanaban M, et al. IP6 is an HIV pocket factor that prevents capsid collapse and promotes DNA synthesis. Elife. 2018;7:e35335. doi: 10.7554/eLife.35335 29848441 PMC6039178

[48] Campbell EM, Hope TJ. HIV-1 capsid: the multifaceted key player in HIV-1 infection. Nat Rev Microbiol. 2015;13(8):471–83. doi: 10.1038/nrmicro3503 26179359 PMC4876022

[49] Bichel K, Price AJ, Schaller T, Towers GJ, Freund SMV, James LC. HIV-1 capsid undergoes coupled binding and isomerization by the nuclear pore protein NUP358. Retrovirology. 2013;10:81. doi: 10.1186/1742-4690-10-81 23902822 PMC3750474

[50] Di Nunzio F, Danckaert A, Fricke T, Perez P, Fernandez J, Perret E, et al. Human nucleoporins promote HIV-1 docking at the nuclear pore, nuclear import and integration. PLoS One. 2012;7(9):e46037. doi: 10.1371/journal.pone.0046037 23049930 PMC3457934

[51] Li W, Singh PK, Sowd GA, Bedwell GJ, Jang S, Achuthan V, et al. CPSF6-dependent targeting of speckle-associated domains distinguishes primate from nonprimate lentiviral integration. mBio. 2020;11(5):e02254-20. doi: 10.1128/mBio.02254-20 32994325 PMC7527728

[52] Freed EO. HIV-1 gag proteins: diverse functions in the virus life cycle. Virology. 1998;251(1):1–15. doi: 10.1006/viro.1998.9398 9813197

[53] Gitti RK, Lee BM, Walker J, Summers MF, Yoo S, Sundquist WI. Structure of the amino-terminal core domain of the HIV-1 capsid protein. Science. 1996;273(5272):231–5. doi: 10.1126/science.273.5272.231 8662505

[54] Ganser-Pornillos BK, Cheng A, Yeager M. Structure of full-length HIV-1 CA: a model for the mature capsid lattice. Cell. 2007;131(1):70–9. doi: 10.1016/j.cell.2007.08.018 17923088

[55] Zhao G, Perilla JR, Yufenyuy EL, Meng X, Chen B, Ning J, et al. Mature HIV-1 capsid structure by cryo-electron microscopy and all-atom molecular dynamics. Nature. 2013;497(7451):643–6. doi: 10.1038/nature12162 23719463 PMC3729984

[56] Takemura T, Murakami T. Functional constraints on HIV-1 capsid: their impacts on the viral immune escape potency. Front Microbiol. 2012;3:369. doi: 10.3389/fmicb.2012.00369 23087682 PMC3474374

[57] von Schwedler UK, Stray KM, Garrus JE, Sundquist WI. Functional surfaces of the human immunodeficiency virus type 1 capsid protein. J Virol. 2003;77(9):5439–50. doi: 10.1128/jvi.77.9.5439-5450.2003 12692245 PMC153941

[58] Gamble TR, Vajdos FF, Yoo S, Worthylake DK, Houseweart M, Sundquist WI, et al. Crystal structure of human cyclophilin A bound to the amino-terminal domain of HIV-1 capsid. Cell. 1996;87(7):1285–94. doi: 10.1016/s0092-8674(00)81823-1 8980234

[59] Franke EK, Yuan HE, Luban J. Specific incorporation of cyclophilin A into HIV-1 virions. Nature. 1994;372(6504):359–62. doi: 10.1038/372359a0 7969494

[60] Padron A, Dwivedi R, Chakraborty R, Prakash P, Kim K, Shi J, et al. Cyclophilin A facilitates HIV-1 integration. J Virol. 2024;98(11):e0094724. doi: 10.1128/jvi.00947-24 39480090 PMC11575316

[61] Matsuoka S, Dam E, Lecossier D, Clavel F, Hance AJ. Modulation of HIV-1 infectivity and cyclophilin A-dependence by Gag sequence and target cell type. Retrovirology. 2009;6:21. doi: 10.1186/1742-4690-6-21 19254360 PMC2653016

[62] De Iaco A, Luban J. Cyclophilin A promotes HIV-1 reverse transcription but its effect on transduction correlates best with its effect on nuclear entry of viral cDNA. Retrovirology. 2014;11:11. doi: 10.1186/1742-4690-11-11 24479545 PMC3916700

[63] Cook M, Freniere C, Wu C, Lozano F, Xiong Y. Structural insights into HIV-2 CA lattice formation and FG-pocket binding revealed by single-particle cryo-EM. Cell Rep. 2025;44(2):115245. doi: 10.1016/j.celrep.2025.115245 39864060 PMC11912512

[64] Mamede JI, Damond F, Bernardo A de, Matheron S, Descamps D, Battini J-L, et al. Cyclophilins and nucleoporins are required for infection mediated by capsids from circulating HIV-2 primary isolates. Sci Rep. 2017;7:45214. doi: 10.1038/srep45214 28345672 PMC5366920

[65] Gamble TR, Yoo S, Vajdos FF, von Schwedler UK, Worthylake DK, Wang H, et al. Structure of the carboxyl-terminal dimerization domain of the HIV-1 capsid protein. Science. 1997;278(5339):849–53. doi: 10.1126/science.278.5339.849 9346481

[66] Tóth F, Kádas J, Mótyán JA, Tőzsér J. Effect of internal cleavage site mutations in human immunodeficiency virus type 1 capsid protein on its structure and function. FEBS Open Bio. 2016;6(8):847–59. doi: 10.1002/2211-5463.12094 27516963 PMC4971840

[67] Kaplan AH, Manchester M, Smith T, Yang YL, Swanstrom R. Conditional human immunodeficiency virus type 1 protease mutants show no role for the viral protease early in virus replication. J Virol. 1996;70(9):5840–4. doi: 10.1128/JVI.70.9.5840-5844.1996 8709202 PMC190600

[68] Nagy K, Young M, Baboonian C, Merson J, Whittle P, Oroszlan S. Antiviral activity of human immunodeficiency virus type 1 protease inhibitors in a single cycle of infection: evidence for a role of protease in the early phase. J Virol. 1994;68(2):757–65. doi: 10.1128/JVI.68.2.757-765.1994 8289379 PMC236512

[69] Du S, Betts L, Yang R, Shi H, Concel J, Ahn J, et al. Structure of the HIV-1 full-length capsid protein in a conformationally trapped unassembled state induced by small-molecule binding. J Mol Biol. 2011;406(3):371–86. doi: 10.1016/j.jmb.2010.11.027 21146540 PMC3194004

[70] Bester SM, Wei G, Zhao H, Adu-Ampratwum D, Iqbal N, Courouble VV, et al. Structural and mechanistic bases for a potent HIV-1 capsid inhibitor. Science. 2020;370(6514):360–4. doi: 10.1126/science.abb4808 33060363 PMC7831379

[71] Blair WS, Pickford C, Irving SL, Brown DG, Anderson M, Bazin R, et al. HIV capsid is a tractable target for small molecule therapeutic intervention. PLoS Pathog. 2010;6(12):e1001220. doi: 10.1371/journal.ppat.1001220 21170360 PMC3000358

[72] Price AJ, Fletcher AJ, Schaller T, Elliott T, Lee K, KewalRamani VN, et al. CPSF6 defines a conserved capsid interface that modulates HIV-1 replication. PLoS Pathog. 2012;8(8):e1002896. doi: 10.1371/journal.ppat.1002896 22956906 PMC3431306

[73] Price AJ, Jacques DA, McEwan WA, Fletcher AJ, Essig S, Chin JW, et al. Host cofactors and pharmacologic ligands share an essential interface in HIV-1 capsid that is lost upon disassembly. PLoS Pathog. 2014;10(10):e1004459. doi: 10.1371/journal.ppat.1004459 25356722 PMC4214760

[74] Rebensburg SV, Wei G, Larue RC, Lindenberger J, Francis AC, Annamalai AS, et al. Sec24C is an HIV-1 host dependency factor crucial for virus replication. Nat Microbiol. 2021;6(4):435–44. doi: 10.1038/s41564-021-00868-1 33649557 PMC8012256

[75] Ganser-Pornillos BK, Yeager M, Sundquist WI. The structural biology of HIV assembly. Curr Opin Struct Biol. 2008;18(2):203–17. doi: 10.1016/j.sbi.2008.02.001 18406133 PMC2819415

[76] Pornillos O, Ganser-Pornillos BK, Yeager M. Atomic-level modelling of the HIV capsid. Nature. 2011;469(7330):424–7. doi: 10.1038/nature09640 21248851 PMC3075868

[77] Meehan AM, Saenz DT, Guevera R, Morrison JH, Peretz M, Fadel HJ, et al. A cyclophilin homology domain-independent role for Nup358 in HIV-1 infection. PLoS Pathog. 2014;10(2):e1003969. doi: 10.1371/journal.ppat.1003969 24586169 PMC3930637

[78] Bocanegra R, Rodríguez-Huete A, Fuertes MÁ, Del Álamo M, Mateu MG. Molecular recognition in the human immunodeficiency virus capsid and antiviral design. Virus Res. 2012;169(2):388–410. doi: 10.1016/j.virusres.2012.06.016 22728445

[79] Engelman A, Cherepanov P. The structural biology of HIV-1: mechanistic and therapeutic insights. Nat Rev Microbiol. 2012;10(4):279–90. doi: 10.1038/nrmicro2747 22421880 PMC3588166

[80] Troyano-Hernáez P, Reinosa R, Holguín Á. HIV capsid protein genetic diversity across HIV-1 variants and impact on new capsid-inhibitor lenacapavir. Front Microbiol. 2022;13:854974. doi: 10.3389/fmicb.2022.854974 35495642 PMC9039614

[81] Robert X, Gouet P. Deciphering key features in protein structures with the new ENDscript server. Nucleic Acids Res. 2014;42(Web Server issue):W320-4. doi: 10.1093/nar/gku316 24753421 PMC4086106

[82] Kiarie IW, Hoffka G, Laporte M, Leyssen P, Neyts J, Tőzsér J, et al. Efficacy of integrase strand transfer inhibitors and the capsid inhibitor lenacapavir against HIV-2, and exploring the effect of raltegravir on the activity of SARS-CoV-2. Viruses. 2024;16(10):1607. doi: 10.3390/v16101607 39459940 PMC11512360

[83] Bester SM, Adu-Ampratwum D, Annamalai AS, Wei G, Briganti L, Murphy BC, et al. Structural and mechanistic bases of viral resistance to hiv-1 capsid inhibitor lenacapavir. mBio. 2022;13(5):e0180422. doi: 10.1128/mbio.01804-22 36190128 PMC9600929

[84] Gres AT, Kirby KA, McFadden WM, Du H, Liu D, Xu C, et al. Multidisciplinary studies with mutated HIV-1 capsid proteins reveal structural mechanisms of lattice stabilization. Nat Commun. 2023;14(1):5614. doi: 10.1038/s41467-023-41197-7 37699872 PMC10497533

[85] Yang R, Shi J, Byeon I-JL, Ahn J, Sheehan JH, Meiler J, et al. Second-site suppressors of HIV-1 capsid mutations: restoration of intracellular activities without correction of intrinsic capsid stability defects. Retrovirology. 2012;9:30. doi: 10.1186/1742-4690-9-30 22515365 PMC3351742

[86] Aiken C, Rousso I. The HIV-1 capsid and reverse transcription. Retrovirology. 2021;18(1):29. doi: 10.1186/s12977-021-00566-0 34563203 PMC8466977

[87] Pennetzdorfer N, Naik V, Demirdjian S, Hendricks MR, Jamieson CS, Perry JK, et al. Lenacapavir treatment-emergent HIV-1 capsid resistance mutations are frequently associated with replication defects. Sci Transl Med. 2026;18(831):eaea0947. doi: 10.1126/scitranslmed.aea0947 41499523

[88] Saito A, Yamashita M. HIV-1 capsid variability: viral exploitation and evasion of capsid-binding molecules. Retrovirology. 2021;18(1):32. doi: 10.1186/s12977-021-00577-x 34702294 PMC8549334

[89] Schiene-Fischer C, Yu C. Receptor accessory folding helper enzymes: the functional role of peptidyl prolyl cis/trans isomerases. FEBS Lett. 2001;495(1–2):1–6. doi: 10.1016/s0014-5793(01)02326-2 11322937

[90] Thali M, Bukovsky A, Kondo E, Rosenwirth B, Walsh CT, Sodroski J, et al. Functional association of cyclophilin A with HIV-1 virions. Nature. 1994;372(6504):363–5. doi: 10.1038/372363a0 7969495

[91] Braaten D, Franke EK, Luban J. Cyclophilin A is required for an early step in the life cycle of human immunodeficiency virus type 1 before the initiation of reverse transcription. J Virol. 1996;70(6):3551–60. doi: 10.1128/JVI.70.6.3551-3560.1996 8648689 PMC190230

[92] Schaller T, Ocwieja KE, Rasaiyaah J, Price AJ, Brady TL, Roth SL, et al. HIV-1 capsid-cyclophilin interactions determine nuclear import pathway, integration targeting and replication efficiency. PLoS Pathog. 2011;7(12):e1002439. doi: 10.1371/journal.ppat.1002439 22174692 PMC3234246

[93] Selyutina A, Persaud M, Simons LM, Bulnes-Ramos A, Buffone C, Martinez-Lopez A, et al. Cyclophilin a prevents HIV-1 restriction in lymphocytes by blocking human trim5α binding to the viral core. Cell Rep. 2020;30(11):3766-3777.e6. doi: 10.1016/j.celrep.2020.02.100 32187548 PMC7363000

[94] Mamede JI, Sitbon M, Battini J-L, Courgnaud V. Heterogeneous susceptibility of circulating SIV isolate capsids to HIV-interacting factors. Retrovirology. 2013;10:77. doi: 10.1186/1742-4690-10-77 23883001 PMC3751554

[95] Shen Q, Kumari S, Xu C, Jang S, Shi J, Burdick RC, et al. The capsid lattice engages a bipartite NUP153 motif to mediate nuclear entry of HIV-1 cores. Proc Natl Acad Sci U S A. 2023;120(13):e2202815120. doi: 10.1073/pnas.2202815120 36943880 PMC10068764

[96] Matreyek KA, Engelman A. The requirement for nucleoporin NUP153 during human immunodeficiency virus type 1 infection is determined by the viral capsid. J Virol. 2011;85(15):7818–27. doi: 10.1128/JVI.00325-11 21593146 PMC3147902

[97] Sowd GA, Serrao E, Wang H, Wang W, Fadel HJ, Poeschla EM, et al. A critical role for alternative polyadenylation factor CPSF6 in targeting HIV-1 integration to transcriptionally active chromatin. Proc Natl Acad Sci U S A. 2016;113(8):E1054-63. doi: 10.1073/pnas.1524213113 26858452 PMC4776470

[98] Achuthan V, Perreira JM, Sowd GA, Puray-Chavez M, McDougall WM, Paulucci-Holthauzen A, et al. Capsid-CPSF6 Interaction licenses nuclear HIV-1 trafficking to sites of viral DNA integration. Cell Host Microbe. 2018;24(3):392-404.e8. doi: 10.1016/j.chom.2018.08.002 30173955 PMC6368089

[99] Stremlau M, Owens CM, Perron MJ, Kiessling M, Autissier P, Sodroski J. The cytoplasmic body component TRIM5alpha restricts HIV-1 infection in Old World monkeys. Nature. 2004;427(6977):848–53. doi: 10.1038/nature02343 14985764

[100] Goujon C, Moncorgé O, Bauby H, Doyle T, Ward CC, Schaller T, et al. Human MX2 is an interferon-induced post-entry inhibitor of HIV-1 infection. Nature. 2013;502(7472):559–62. doi: 10.1038/nature12542 24048477 PMC3808269

[101] Fricke T, White TE, Schulte B, de Souza Aranha Vieira DA, Dharan A, Campbell EM, et al. MxB binds to the HIV-1 core and prevents the uncoating process of HIV-1. Retrovirology. 2014;11:68. doi: 10.1186/s12977-014-0068-x 25123063 PMC4145229

[102] Yufenyuy EL, Aiken C. The NTD-CTD intersubunit interface plays a critical role in assembly and stabilization of the HIV-1 capsid. Retrovirology. 2013;10:29. doi: 10.1186/1742-4690-10-29 23497318 PMC3623829

[103] Gilead Sciences I. Sunlenca® (lenacapavir) receives fda approval as a first-in-class, twice-yearly treatment option for people living with multi-drug resistant hiv. Available from: https://www.gilead.com/news/news-details/2022/sunlenca-lenacapavir-receives-fda-approval-as-a-first-in-class-twice-yearly-treatment-option-for-people-living-with-multi-drug-resistant-hiv. Accessed 2025 June 10.

[104] Segal-Maurer S, DeJesus E, Stellbrink H-J, Castagna A, Richmond GJ, Sinclair GI, et al. Capsid inhibition with lenacapavir in multidrug-resistant HIV-1 infection. N Engl J Med. 2022;386(19):1793–803. doi: 10.1056/NEJMoa2115542 35544387

[105] Huang S-W, Briganti L, Annamalai AS, Greenwood J, Shkriabai N, Haney R, et al. The primary mechanism for highly potent inhibition of HIV-1 maturation by lenacapavir. PLoS Pathog. 2025;21(1):e1012862. doi: 10.1371/journal.ppat.1012862 39869652 PMC11892807

[106] Link JO, Rhee MS, Tse WC, Zheng J, Somoza JR, Rowe W, et al. Clinical targeting of HIV capsid protein with a long-acting small molecule. Nature. 2020;584(7822):614–8. doi: 10.1038/s41586-020-2443-1 32612233 PMC8188729

[107] Yant SR, Mulato A, Hansen D, Tse WC, Niedziela-Majka A, Zhang JR, et al. A highly potent long-acting small-molecule HIV-1 capsid inhibitor with efficacy in a humanized mouse model. Nat Med. 2019;25(9):1377–84. doi: 10.1038/s41591-019-0560-x 31501601 PMC7396128

[108] Zheng J, Yant SR, Ahmadyar S, Chan TY, Chiu A, Cihlar T, et al. 539. GS-CA2: a novel, potent, and selective first-in-class inhibitor of HIV-1 capsid function displays nonclinical pharmacokinetics supporting long-acting potential in humans. Open Forum Infect Dis. 2018;5(suppl_1):S199–200. doi: 10.1093/ofid/ofy210.548

[109] Tse W, Link J, Mulato A, Niedziela-Majka A, Rowe W, Somoza J, et al. Discovery of novel potent HIV capsid inhibitors with long-acting potential. In: Conference on retroviruses and opportunistic infections. Seattle, Washington, 2017. pp. 13–6.

[110] Vidal SJ, Bekerman E, Hansen D, Lu B, Wang K, Mwangi J, et al. Long-acting capsid inhibitor protects macaques from repeat SHIV challenges. Nature. 2022;601(7894):612–6. doi: 10.1038/s41586-021-04279-4 34875675 PMC8753592

[111] Lamorte L, Titolo S, Lemke CT, Goudreau N, Mercier J-F, Wardrop E, et al. Discovery of novel small-molecule HIV-1 replication inhibitors that stabilize capsid complexes. Antimicrob Agents Chemother. 2013;57(10):4622–31. doi: 10.1128/AAC.00985-13 23817385 PMC3811413

[112] Tang C, Loeliger E, Kinde I, Kyere S, Mayo K, Barklis E, et al. Antiviral inhibition of the HIV-1 capsid protein. J Mol Biol. 2003;327(5):1013–20. doi: 10.1016/s0022-2836(03)00289-4 12662926

[113] Kelly BN, Kyere S, Kinde I, Tang C, Howard BR, Robinson H, et al. Structure of the antiviral assembly inhibitor CAP-1 complex with the HIV-1 CA protein. J Mol Biol. 2007;373(2):355–66. doi: 10.1016/j.jmb.2007.07.070 17826792 PMC2066180

[114] Laskowski RA, Swindells MB. Ligplot : multiple ligand-protein interaction diagrams for drug discovery. J Chem Inf Model. 2011;51:2778–86. 21919503 10.1021/ci200227u

[115] Corso GHS, Jing B, Barzilay R, Jaakkola T. Diffdock: diffusion steps, twists, and turns for molecular docking. arXiv. 2023.

[116] Abramson J, Adler J, Dunger J, Evans R, Green T, Pritzel A, et al. Accurate structure prediction of biomolecular interactions with AlphaFold 3. Nature. 2024;630(8016):493–500. doi: 10.1038/s41586-024-07487-w 38718835 PMC11168924

[117] Lemke CT, Titolo S, von Schwedler U, Goudreau N, Mercier J-F, Wardrop E, et al. Distinct effects of two HIV-1 capsid assembly inhibitor families that bind the same site within the N-terminal domain of the viral CA protein. J Virol. 2012;86(12):6643–55. doi: 10.1128/JVI.00493-12 22496222 PMC3393593

[118] Pornillos O, Ganser-Pornillos BK, Kelly BN, Hua Y, Whitby FG, Stout CD, et al. X-ray structures of the hexameric building block of the HIV capsid. Cell. 2009;137(7):1282–92. doi: 10.1016/j.cell.2009.04.063 19523676 PMC2840706

[119] Kortagere S, Xu JP, Mankowski MK, Ptak RG, Cocklin S. Structure-activity relationships of a novel capsid targeted inhibitor of HIV-1 replication. J Chem Inf Model. 2014;54(11):3080–90. doi: 10.1021/ci500437r 25302989 PMC4245176

[120] Kortagere S, Madani N, Mankowski MK, Schön A, Zentner I, Swaminathan G, et al. Inhibiting early-stage events in HIV-1 replication by small-molecule targeting of the HIV-1 capsid. J Virol. 2012;86(16):8472–81. doi: 10.1128/JVI.05006-11 22647699 PMC3421734

[121] Sticht J, Humbert M, Findlow S, Bodem J, Müller B, Dietrich U, et al. A peptide inhibitor of HIV-1 assembly in vitro. Nat Struct Mol Biol. 2005;12(8):671–7. doi: 10.1038/nsmb964 16041387

[122] Zhang H, Zhao Q, Bhattacharya S, Waheed AA, Tong X, Hong A, et al. A cell-penetrating helical peptide as a potential HIV-1 inhibitor. J Mol Biol. 2008;378(3):565–80. doi: 10.1016/j.jmb.2008.02.066 18374356 PMC2695608

[123] Zhang H, Curreli F, Waheed AA, Mercredi PY, Mehta M, Bhargava P, et al. Dual-acting stapled peptides target both HIV-1 entry and assembly. Retrovirology. 2013;10:136. doi: 10.1186/1742-4690-10-136 24237936 PMC3842668

[124] Wang Y, Curreli F, Xu WS, Li ZP, Kong DS, Ren L, et al. Antiviral activity of dual-acting hydrocarbon-stapled peptides against HIV-1 predominantly circulating in China. Biomed Environ Sci. 2017;30(6):398–406. doi: 10.3967/bes2017.053 28705263

[125] Thenin-Houssier S, Valente ST. HIV-1 capsid inhibitors as antiretroviral agents. Curr HIV Res. 2016;14(3):270–82. doi: 10.2174/1570162x14999160224103555 26957201 PMC4785820

[126] Garzón MT, Lidón-Moya MC, Barrera FN, Prieto A, Gómez J, Mateu MG, et al. The dimerization domain of the HIV-1 capsid protein binds a capsid protein-derived peptide: a biophysical characterization. Protein Sci. 2004;13(6):1512–23. doi: 10.1110/ps.03555304 15152086 PMC2279969

[127] Bocanegra R, Nevot M, Doménech R, López I, Abián O, Rodríguez-Huete A, et al. Rationally designed interfacial peptides are efficient in vitro inhibitors of HIV-1 capsid assembly with antiviral activity. PLoS One. 2011;6(9):e23877. doi: 10.1371/journal.pone.0023877 21931621 PMC3169566

[128] Nozawa R, Yokota T, Fujimoto T. Susceptibility of methicillin-resistant *Staphylococcus aureus* to the selenium-containing compound 2-phenyl-1,2-benzoisoselenazol-3(2h)-one (pz51). Antimicrob Agents Chemother. 1989;33:1388–90. 2802564 10.1128/aac.33.8.1388 PMC172662

[129] Maślanka M, Mucha A. Antibacterial activity of ebselen. Int J Mol Sci. 2023;24(2):1610. doi: 10.3390/ijms24021610 36675123 PMC9864093

[130] Thenin-Houssier S, de Vera IMS, Pedro-Rosa L, Brady A, Richard A, Konnick B, et al. Ebselen, a small-molecule capsid inhibitor of HIV-1 replication. Antimicrob Agents Chemother. 2016;60(4):2195–208. doi: 10.1128/AAC.02574-15 26810656 PMC4808204

[131] Zhang D-W, Yan H-L, Xu X-S, Xu L, Yin Z-H, Chang S, et al. The selenium-containing drug ebselen potently disrupts LEDGF/p75-HIV-1 integrase interaction by targeting LEDGF/p75. J Enzyme Inhib Med Chem. 2020;35(1):906–12. doi: 10.1080/14756366.2020.1743282 32228103 PMC7170385

[132] Azad GK, Singh V, Mandal P, Singh P, Golla U, Baranwal S, et al. Ebselen induces reactive oxygen species (ROS)-mediated cytotoxicity in *Saccharomyces cerevisiae* with inhibition of glutamate dehydrogenase being a target. FEBS Open Bio. 2014;4:77–89. doi: 10.1016/j.fob.2014.01.002 24490132 PMC3907691

[133] Boulay A, Quevarec E, Malet I, Nicastro G, Chamontin C, Perrin S, et al. A new class of capsid-targeting inhibitors that specifically block HIV-1 nuclear import. EMBO Mol Med. 2024;16(11):2918–45. doi: 10.1038/s44321-024-00143-w 39358603 PMC11555092

[134] Mann A, Friedrich N, Krarup A, Weber J, Stiegeler E, Dreier B, et al. Conformation-dependent recognition of HIV gp120 by designed ankyrin repeat proteins provides access to novel HIV entry inhibitors. J Virol. 2013;87(10):5868–81. doi: 10.1128/JVI.00152-13 23487463 PMC3648163

[135] Stumpp MT, Binz HK, Amstutz P. DARPins: a new generation of protein therapeutics. Drug Discov Today. 2008;13(15–16):695–701. doi: 10.1016/j.drudis.2008.04.013 18621567

[136] Schweizer A, Rusert P, Berlinger L, Ruprecht CR, Mann A, Corthésy S, et al. CD4-specific designed ankyrin repeat proteins are novel potent HIV entry inhibitors with unique characteristics. PLoS Pathog. 2008;4(7):e1000109. doi: 10.1371/journal.ppat.1000109 18654624 PMC2453315

[137] Nangola S, Urvoas A, Valerio-Lepiniec M, Khamaikawin W, Sakkhachornphop S, Hong S-S, et al. Antiviral activity of recombinant ankyrin targeted to the capsid domain of HIV-1 Gag polyprotein. Retrovirology. 2012;9:17. doi: 10.1186/1742-4690-9-17 22348230 PMC3308923

[138] Saoin S, Wisitponchai T, Intachai K, Chupradit K, Moonmuang S, Nangola S, et al. Deciphering critical amino acid residues to modify and enhance the binding affinity of ankyrin scaffold specific to capsid protein of human immunodeficiency virus type 1. Asian Pac J Allergy Immunol. 2018;36(2):126–35. doi: 10.12932/AP-280217-0037 28802032

[139] Juntit O-A, Sornsuwan K, Wisitponchai T, Sanghiran Lee V, Sakkhachornphop S, Yasamut U, et al. Dimeric ankyrin with inverted module promotes bifunctional property in capturing capsid to impede HIV-1 replication. Int J Mol Sci. 2023;24(6):5266. doi: 10.3390/ijms24065266 36982337 PMC10048781

[140] Wang C, Huang H, Mallon K, Valera L, Parcella K, Cockett MI, et al. Antiviral properties of HIV-1 capsid inhibitor GSK878. Antimicrob Agents Chemother. 2023;67(5):e0169422. doi: 10.1128/aac.01694-22 37039636 PMC10190262

[141] Thakkar N, Griesel R, Pierce A, Bainbridge V, Shepherd B, Angelis K, et al. Clinical Pharmacokinetics and safety of a new HIV-1 capsid inhibitor, VH4004280, after oral administration in adults without HIV. Infect Dis Ther. 2025;14(6):1313–26. doi: 10.1007/s40121-025-01154-x 40287607 PMC12151970

[142] Wang C, Huang H, Valera L, Parcella K, Iwuagwu C, McAuliffe B, et al. Preclinical virology profiles of the HIV-1 capsid inhibitors VH4004280 and VH4011499. Antimicrob Agents Chemother. 2025;69(10):e0030925. doi: 10.1128/aac.00309-25 40899688 PMC12486825

[143] Nka AD, Bouba Y, Teto G, Semengue ENJ, Takou DK, Ngueko AMK, et al. Evaluation of HIV-1 capsid genetic variability and lenacapavir (GS-6207) drug resistance-associated mutations according to viral clades among drug-naive individuals. J Antimicrob Chemother. 2022;78(1):272–5. doi: 10.1093/jac/dkac388 36411257

[144] Tao K, Rhee S-Y, Tzou PL, Osman ZA, Pond SLK, Holmes SP, et al. HIV-1 Group M capsid amino acid variability: implications for sequence quality control of genotypic resistance testing. Viruses. 2023;15(4):992. doi: 10.3390/v15040992 37112972 PMC10143361

[145] Choudhary MC, Li JZ. When HIV pays the price: fitness costs behind lenacapavir resistance. Sci Transl Med. 2026;18(831):eaed6475. doi: 10.1126/scitranslmed.aed6475 41499521

[146] McFadden WM, Snyder AA, Kirby KA, Tedbury PR, Raj M, Wang Z, et al. Rotten to the core: antivirals targeting the HIV-1 capsid core. Retrovirology. 2021;18(1):41. doi: 10.1186/s12977-021-00583-z 34937567 PMC8693499

[147] Zhou J, Price AJ, Halambage UD, James LC, Aiken C. HIV-1 resistance to the capsid-targeting inhibitor PF74 results in altered dependence on host factors required for virus nuclear entry. J Virol. 2015;89(17):9068–79. doi: 10.1128/JVI.00340-15 26109731 PMC4524096

[148] Carnes SK, Sheehan JH, Aiken C. Inhibitors of the HIV-1 capsid, a target of opportunity. Curr Opin HIV AIDS. 2018;13(4):359–65. doi: 10.1097/COH.0000000000000472 29782334 PMC6075716

[149] Shi J, Zhou J, Halambage UD, Shah VB, Burse MJ, Wu H, et al. Compensatory substitutions in the HIV-1 capsid reduce the fitness cost associated with resistance to a capsid-targeting small-molecule inhibitor. J Virol. 2015;89(1):208–19. doi: 10.1128/JVI.01411-14 25320302 PMC4301104

[150] Ogbuagu O, McGowan JP, Stapleton A, Wiznia A, Berger D, Creticos CM, et al. Long-acting subcutaneous lenacapavir in people with multi-drug resistant hiv-1: 3-year results of the capella study. Open Forum Infect Dis. 2025;12. 10.1093/ofid/ofaf763 PMC12740718 41459297

[151] Gupta SK, Berhe M, Crofoot G, Benson P, Ramgopal M, Sims J, et al. Lenacapavir administered every 26 weeks or daily in combination with oral daily antiretroviral therapy for initial treatment of HIV: a randomised, open-label, active-controlled, phase 2 trial. Lancet HIV. 2023;10(1):e15–23. doi: 10.1016/S2352-3018(22)00291-0 36566079

[152] Selzer L, VanderVeen LA, Parvangada A, Martin R, Collins SE, Mehrotra M, et al. Susceptibility screening of HIV-1 viruses to broadly neutralizing antibodies, teropavimab and zinlirvimab, in people with HIV-1 suppressed by antiretroviral therapy. J Acquir Immune Defic Syndr. 2025;98(1):64–71. doi: 10.1097/QAI.0000000000003528 39298557

[153] Eron JJ, Little SJ, Crofoot G, Cook P, Ruane PJ, Jayaweera D, et al. Safety of teropavimab and zinlirvimab with lenacapavir once every 6 months for HIV treatment: a phase 1b, randomised, proof-of-concept study. Lancet HIV. 2024;11(3):e146–55. doi: 10.1016/S2352-3018(23)00293-X 38307098 PMC12245405

[154] Kelley CF, Acevedo-Quiñones M, Agwu AL, Avihingsanon A, Benson P, Blumenthal J, et al. Twice-yearly lenacapavir for hiv prevention in men and gender-diverse persons. N Engl J Med. 2025;392(13):1261–76. doi: 10.1056/NEJMoa2411858 39602624

[155] Bekker L-G, Das M, Abdool Karim Q, Ahmed K, Batting J, Brumskine W, et al. Twice-yearly lenacapavir or daily F/TAF for HIV prevention in cisgender women. N Engl J Med. 2024;391(13):1179–92. doi: 10.1056/NEJMoa2407001 39046157

[156] Lepik KJ, Harrigan PR, Yip B, Wang L, Robbins MA, Zhang WW, et al. Emergent drug resistance with integrase strand transfer inhibitor-based regimens. AIDS. 2017;31(10):1425–34. doi: 10.1097/QAD.0000000000001494 28375875

[157] Margot NA, Jogiraju V, Pennetzdorfer N, Naik V, VanderVeen LA, Ling J, et al. Resistance analyses in heavily treatment-experienced people with HIV treated with the novel HIV capsid inhibitor lenacapavir after 2 years. J Infect Dis. 2025;231(5):1239–45. doi: 10.1093/infdis/jiaf050 39873394

[158] Tanaka K, Kim Y, Roche M, Lewin SR. The role of latency reversal in HIV cure strategies. J Med Primatol. 2022;51(5):278–83. doi: 10.1111/jmp.12613 36029233 PMC9514955

[159] McFadden WM, Faerch M, Kirby KA, Dick RA, Torbett BE, Sarafianos SG. Considerations for capsid-targeting antiretrovirals in pre-exposure prophylaxis. Trends Mol Med. 2025;31(9):801–13. doi: 10.1016/j.molmed.2025.01.013 40021388 PMC12270248

[160] Scanlon ML, Vreeman RC. Current strategies for improving access and adherence to antiretroviral therapies in resource-limited settings. HIV AIDS (Auckl). 2013;5:1–17. doi: 10.2147/HIV.S28912 23326204 PMC3544393

[161] Oturu K, O’Brien O, Ozo-Eson PI. Barriers and enabling structural forces affecting access to antiretroviral therapy in Nigeria. BMC Public Health. 2024;24(1):105. doi: 10.1186/s12889-023-17271-6 38184516 PMC10770989

[162] Cohen J. A bloodbath’: Hiv field is reeling after billions in u.S. funding are axed. ScienceInsider. 2025.

